# Analysis and Refinement of Host–Guest Interactions in Metal–Organic Frameworks

**DOI:** 10.1021/acs.accounts.3c00243

**Published:** 2023-08-30

**Authors:** Yinlin Chen, Wanpeng Lu, Martin Schröder, Sihai Yang

**Affiliations:** † Department of Chemistry, University of Manchester, Manchester M13 9PL, U.K.; ‡ College of Chemistry and Molecular Engineering, Beijing National Laboratory for Molecular Sciences, Peking University, Beijing 100871, China

## Abstract

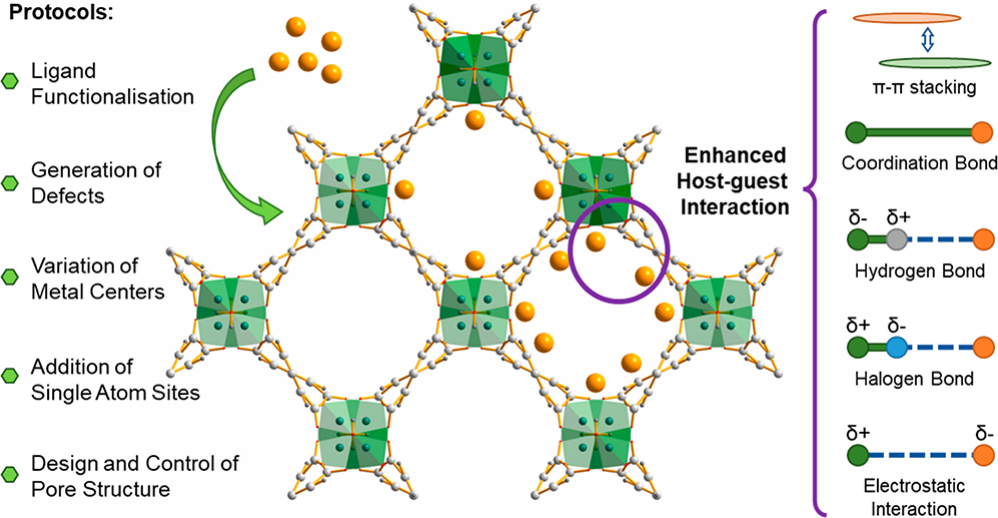

Metal–organic frameworks (MOFs) are a class of hybrid porous materials characterized by their periodic assembly using metal ions and organic ligands through coordination bonds. Their high crystallinity, extensive surface area, and adjustable pore sizes make them promising candidates for a wide array of applications. These include gas adsorption and separation, substrate binding, and catalysis, of relevance to tackling pressing global issues such as climate change, energy challenges, and pollution. In comparison to traditional porous materials such as zeolites and activated carbons, the design flexibility of organic ligands in MOFs, coupled with their orderly arrangement with associated metal centers, allows for the precise engineering of uniform pore environments. This unique feature enables a rich variety of interactions between the MOF host and adsorbed gas molecules, which are fundamental to understanding the observed uptake capacity and selectivity for target gas molecules and thus the overall performance of the material.

In this Account, a data set for three-dimensional MOFs has been constructed based upon the structural analysis of host–guest interactions using the largest experimental database, the Cambridge Structural Database (CSD). A full screening was performed on structures with guest molecules of H_2_, C_2_H_2_, CO_2_, and SO_2_, and the relationship between the primary binding site, the isosteric heats of adsorption (*Q*_st_), and the adsorption uptake was extracted and established. We review the methodologies to refine host–guest interactions based primarily on our studies on the host–guest chemistry of MOFs. The methods include ligand functionalization, variation of metal centers, formation of defects, addition of single atom sites, and control of pore size and structure. *In situ* structural and dynamic investigations using diffraction and spectroscopic techniques are powerful tools to visualize the details of host–guest interactions upon the above modifications, affording key insights into functional performance at a molecular level. Finally, we give an outlook of future research priorities in the study of host–guest chemistry in MOF materials. We hope this Account will encourage the rational development and improvement of future MOF-based sorbents for applications in challenging gas adsorption, separations, and catalysis.

## Key References

- LuZ.; GodfreyH. G. W.; da SilvaI.; ChengY.; SavageM.; TunaF.; McInnesE. J. L.; TeatS. J.; GagnonK. J.; FrogleyM. D.; ManuelP.; RudićS.; Ramirez-CuestaA. J.; EasunT. L.; YangS.; SchröderM.Modulating Supramolecular Binding of Carbon Dioxide in a Redox-Active Porous Metal–Organic Framework. Nat. Commun.2017, 8, 1421210.1038/ncomms1421228194014PMC5316804.^1^*Variation of the oxidation state of metal centers in MOFs has a notable impact on host–guest interactions, thus influencing the adsorption of CO*_2_.
- SmithG. L.; EyleyJ. E.; HanX.; ZhangX.; LiJ.; JacquesN. M.; GodfreyH. G. W.; ArgentS. P.; McCormick McPhersonL. J.; TeatS. J.; ChengY.; FrogleyM. D.; CinqueG.; DayS. J.; TangC. C.; EasunT. L.; RudićS.; Ramirez-CuestaA. J.; YangS.; SchröderM.Reversible Coordinative Binding and Separation of Sulfur Dioxide in a Robust Metal–Organic Framework with Open Copper Sites. Nat. Mater.2019, 18, 1358–136510.1038/s41563-019-0495-031611671.^2^*A benchmark material for SO*_*2*_*adsorption is reported, where the role of open metal sites is unambiguously revealed by the direct observation of coordinative binding as well as in control experiments*.
- LiW.; LiJ.; DuongT. D.; SapchenkoS. A.; HanX.; HumbyJ. D.; WhiteheadG. F. S.; Victórica-YrezábalI. J.; da SilvaI.; ManuelP.; FrogleyM. D.; CinqueG.; SchröderM.; YangS.Adsorption of Sulfur Dioxide in Cu(II)-Carboxylate Framework Materials: The Role of Ligand Functionalization and Open Metal Sites. J. Am. Chem. Soc.2022, 144, 13196–1320410.1021/jacs.2c0328035848823PMC9345647.^3^*The influence of various functional groups on the adsorption mechanism of SO*_*2*_*in MOFs has been systematically studied*.
- AnB.; LiZ.; WangZ.; ZengX.; HanX.; ChengY.; ShevelevaA. M.; ZhangZ.; TunaF.; McInnesE. J. L.; FrogleyM. D.; Ramirez-CuestaA. J.; NatrajanL. S.; WangC.; LinW.; YangS.; SchröderM.Direct Photo-oxidation of Methane to Methanol over a Mono-iron Hydroxyl Site. Nat. Mater.2022, 21, 932–93810.1038/s41563-022-01279-135773491.^4^*Confined adsorption of methane (CH*_*4*_*) over monoiron hydroxyl sites immobilized within a metal–organic framework through linker modification promotes direct photo-oxidation of CH*_*4*_*to CH*_3_*OH*.

## 1 Introduction

The recent development of MOFs has greatly advanced the chemistry and applications of porous materials. With exceptional surface area (up to 8000 m^2^ g^–1^), adjustable pore size, tunable functionality, and design flexibility, MOFs have been studied extensively for various applications, such as gas adsorption and separation,^5,6^ sensing,^7^ proton conductivity,^8^ drug delivery,^9^ and catalysis.^10^ Gas adsorption and separation using MOFs have shown great potential to address many challenging environmental problems, such as global warming, air pollution, and the demand for clean energy.^11^

Surface area and pore chemistry often serve as fundamental factors for evaluating the gas adsorption performance of MOFs.^12^ In porous solids, higher values of pore volume and/or surface area are generally considered indicators of the potential for higher adsorption capacities, particularly for adsorption under (near) saturation conditions. In contrast, adsorption behavior in the low-pressure regime is primarily influenced by host–guest interactions, the strength of which is typically reflected by the isosteric heats of adsorption (*Q*_st_) and the Henry’s constant. The impact of these interactions is pronounced in (ultra) microporous materials, where the pore can accommodate only a few layers of adsorbates. Thus, refining the porosity as well as pore interior, such as the presence of functional groups and/or open metal sites, has been a common approach to improve the performance of adsorption and separation in MOFs.^13,14^ Computational studies on the modeling of host–guest interactions in MOFs have also emerged.^15^ It is therefore timely to review the available tools to refine the host–guest interactions in MOFs to deliver the desired adsorption performance.

In this Account, we analyze the experimental structural information within the Cambridge Structure Database (CSD) to derive additional insights into the relationships between adsorption performance and observed host–guest interactions in MOFs. Such a structural perspective offers critical information about interaction and binding preferences. We then discuss the key research progress originating from our research on the refinement of host–guest interactions to achieve enhanced adsorption performance in MOFs. Through the control of the pore chemistry via ligand functionalization, variation of metal centers, formation of defects, addition of single atom sites, and control of pore structure, significant progress has been achieved in the development of porous MOF materials for the adsorption of a wide range of gases and volatile organic compounds. We hope that this Account will encourage the rational development and improvement of future MOF-based sorbents for applications in challenging gas adsorption, separations, and catalysis.

### 1.1 Construction of MOF Data Set

Advanced X-ray and neutron powder diffraction (NPD) techniques have been employed to study host–guest interactions in MOFs. These enable direct visualization of guest molecules within MOF pores at the molecular level, thus defining and determining preferred binding sites and predominant interactions.^16^ Previously reported databases of MOFs have shown great success in large-scale screening of MOF materials for target properties, such as H_2_ storage,^17^ CH_4_ storage,^18^ and CO_2_ capture.^19,20^ These databases are constructed based on either experimental data [CoRE,^21^ CSD MOF subset^22^] or hypothetical models [hMOF,^23^ ToBaCCo^24^]. Here, to extract host–guest structural information, we developed a new top-down method to search for MOFs containing guest molecules from the CSD database. According to the definition by the International Union of Pure and Applied Chemistry (IUPAC),^25^ a MOF is a coordination network with organic ligands containing potential voids, where the network expands in two or three dimensions. Of the total 1.18 million structures in the v5.43 CSD database, 686 000 entries contain metal elements, among which 200 000 entries have coordination bonds to organic ligands. There are 40 839 structures of coordinated compounds that can expand throughout the entire space. After neutral guest molecules and monodentate ligands (typically solvent molecules) attached to the framework were eliminated, 33 931 entries in the database have potential voids using a “probe” with a radius of 1.2 Å and were collected into our MOF data set. Additional details are given in the Supporting Information.

### 1.2 Analysis of Host–Guest Interactions

The most commonly observed neutral guest molecules and monodentate compounds are solvent molecules such as water, dimethylformamide, and methanol derived from the synthesis of the material. This is very common within our MOF data set and is observed in 12 156 cases. An examination of gas-loaded MOF structures that are linked to gas adsorption studies confirms that those containing CO_2_, CH_4_, H_2_, C_2_H_2_, and SO_2_ are prevalent and are thus used in this study. Apart from SO_2_, each gas has more than 30 entries, supporting the rigor and reliability of this analysis.

High Brunauer–Emmett–Teller (BET) surface area is one of the key features of MOFs, which can greatly influence the adsorption performance. The linear correlation between the maximum excess H_2_ adsorption at 77 K and the BET surface area is well documented as Chahine’s rule, with every 1 wt % of adsorption uptake corresponding to 500 m^2^ of the surface area.^26,27^ A similar relationship is established for the adsorption of CH_4_.^28−30^

**Figure 1:**
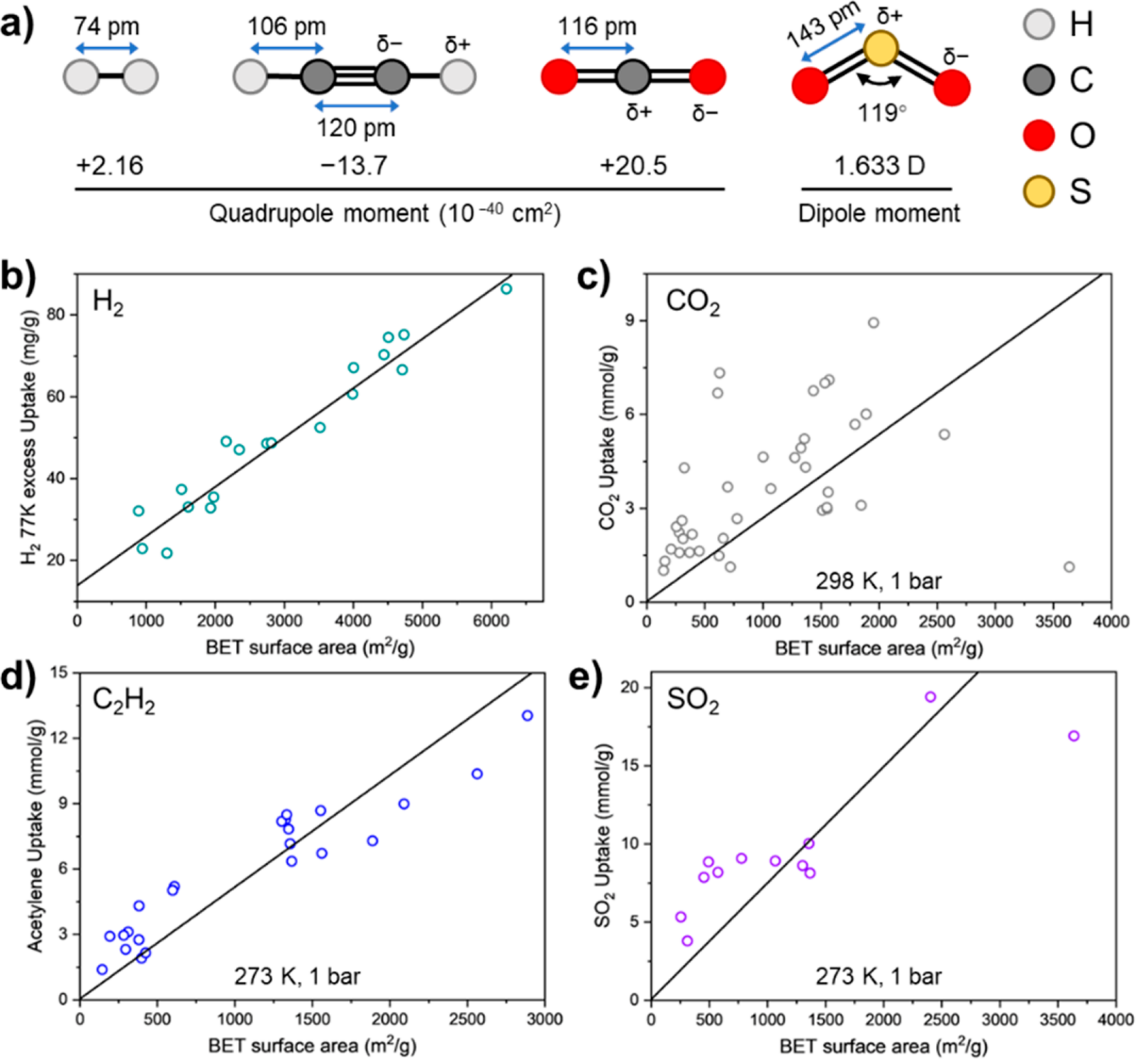
**a**) View of H_2_, CO_2_, C_2_H_2_, and SO_2_ molecules and their dipole/quadrupole moments. (**b**–**e**) Plots of BET surface area vs the uptake of H_2_ [**b**, data obtained from ref (26)], CO_2_ (**c**), C_2_H_2_ (**d**), and SO_2_ (**e**) with Pearson correlation coefficients of 0.979, 0.819, 0.974, and 0.932, respectively.

Utilizing the cases from the compiled data set in this Account, the relationship between the BET surface area and the uptake of the three other more polar gases (C_2_H_2_, CO_2_, and SO_2_) has been appraised. The BET surface area, adsorption performance, *Q*_st_, and host–guest interaction have been extracted from the pertinent structural cases (Tables S2–S6). In contrast to H_2_ and CH_4_, the relationship between the BET surface area and the uptake of C_2_H_2_, CO_2_, and SO_2_ does not demonstrate clear linearity, particularly in the case of C_2_H_2_ and SO_2_ (Figure 1). Linear estimation underestimates the uptake for materials with low BET surface areas and overestimates that for materials with high surface areas, thus an S-shaped curve is obtained as the most accurate descriptor. This is because more substantial host–guest interactions are anticipated in (ultra) microporous materials compared with those showing high porosity. Interestingly, the extent of the deviation of the curve from linearity corresponds to the average strength of host–guest interactions: 10.5 ± 1.2 kJ mol^–1^ for H_2_, 31.0 ± 1.2 kJ mol^–1^ for CO_2_, 43.0 ± 2.7 kJ mol^–1^ for C_2_H_2_, and 49.3 ± 5.4 kJ mol^–1^ for SO_2_ (Table S2, S4, S5, S6).

**Figure 2:**
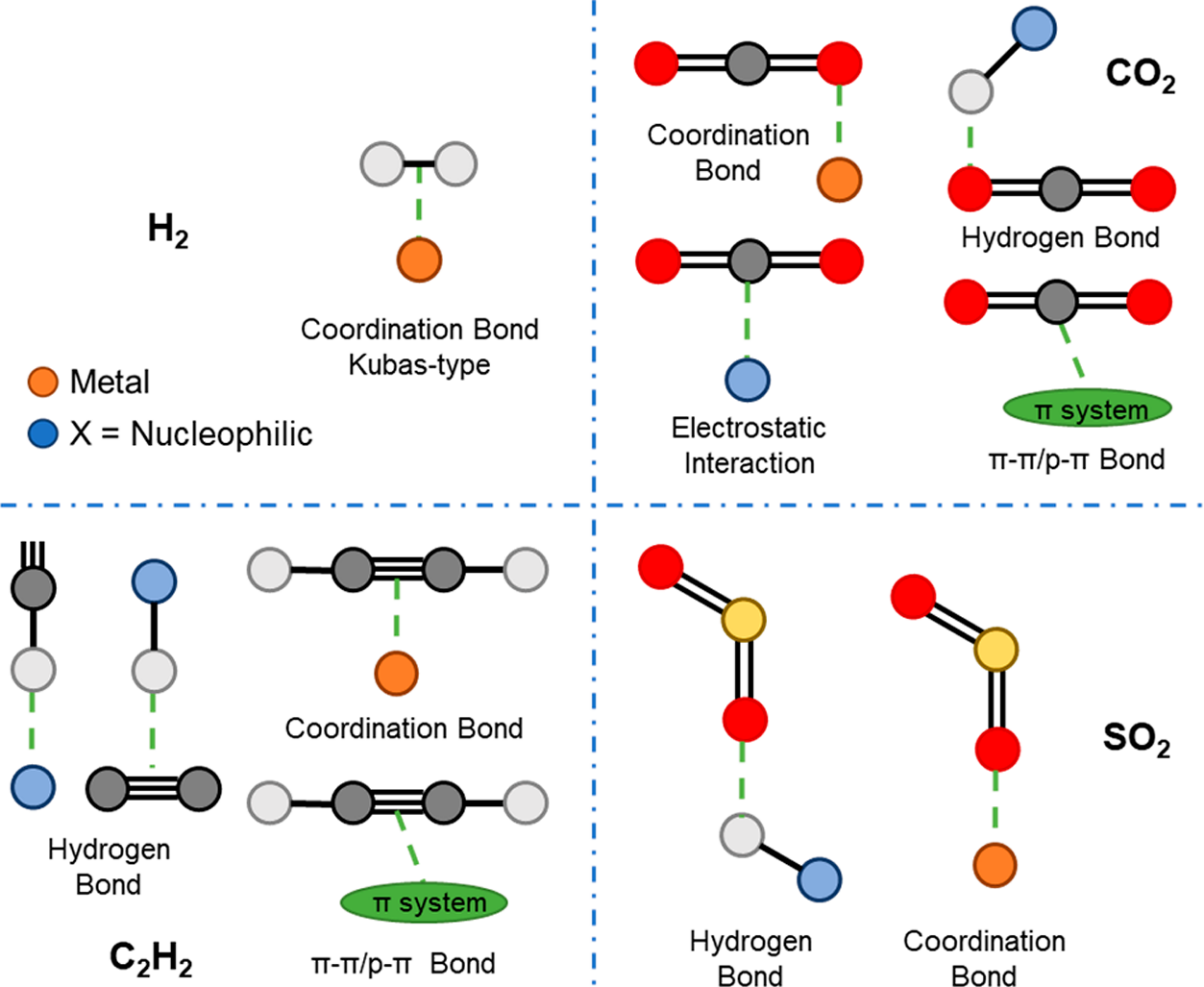
Key interactions of MOFs with adsorbed H_2_, CO_2_, C_2_H_2_, and SO_2_ as observed in the MOF data set.

Further analysis has been conducted to identify the most significant contribution to host–guest interactions in the MOF data set (Figure 2). Coordination bonds appear to govern the host–guest interaction for H_2_ adsorption. In the case of CO_2_, four primary types of interactions, including hydrogen bonding, electrostatic interaction with charged compounds, coordination bonds, and p−π interaction, can significantly influence the host–guest interaction. For C_2_H_2_, hydrogen bonding and π–π interactions constitute the majority of the interactions. Hydrogen and coordination bonding are commonly observed to stabilize adsorbed SO_2_ molecules in MOFs. Thus, molecules possessing greater complexity can afford increased versatility and possibility in forming host–guest interactions, thereby enhancing the gas uptake, particularly at low pressures.

## 2 Refinement of Host–Guest Interactions in MOFs

To date, multiple strategies have been reported to maximize and control the host–guest interactions in MOFs to achieve the desired gas adsorption properties. These include ligand functionalization, variation of metal centers, generation of defects, introduction of single atom sites, and design and control of pore structure, which are discussed in this Account.

### 2.1 Ligand Functionalization

Ligand functionalization is a versatile strategy to tune host–guest interactions in MOFs, benefiting from the abundant tools available in organic chemistry and reticular chemistry.^31^ Functional groups used for ligand decoration range from simple halogen, amino, hydroxyl, and nitro groups to more complex carboxylic acid, sulfonic acid, and metal coordination composites. Postsynthetic protocols offer further possibilities for functionalization in MOFs.^32−34^ Changes in the surface properties are often accompanied by variations in porosity and pore geometry in the resultant MOFs due to steric and conformational effects, which must also be taken into consideration in evaluating and analyzing gas adsorption.

**Figure 3:**
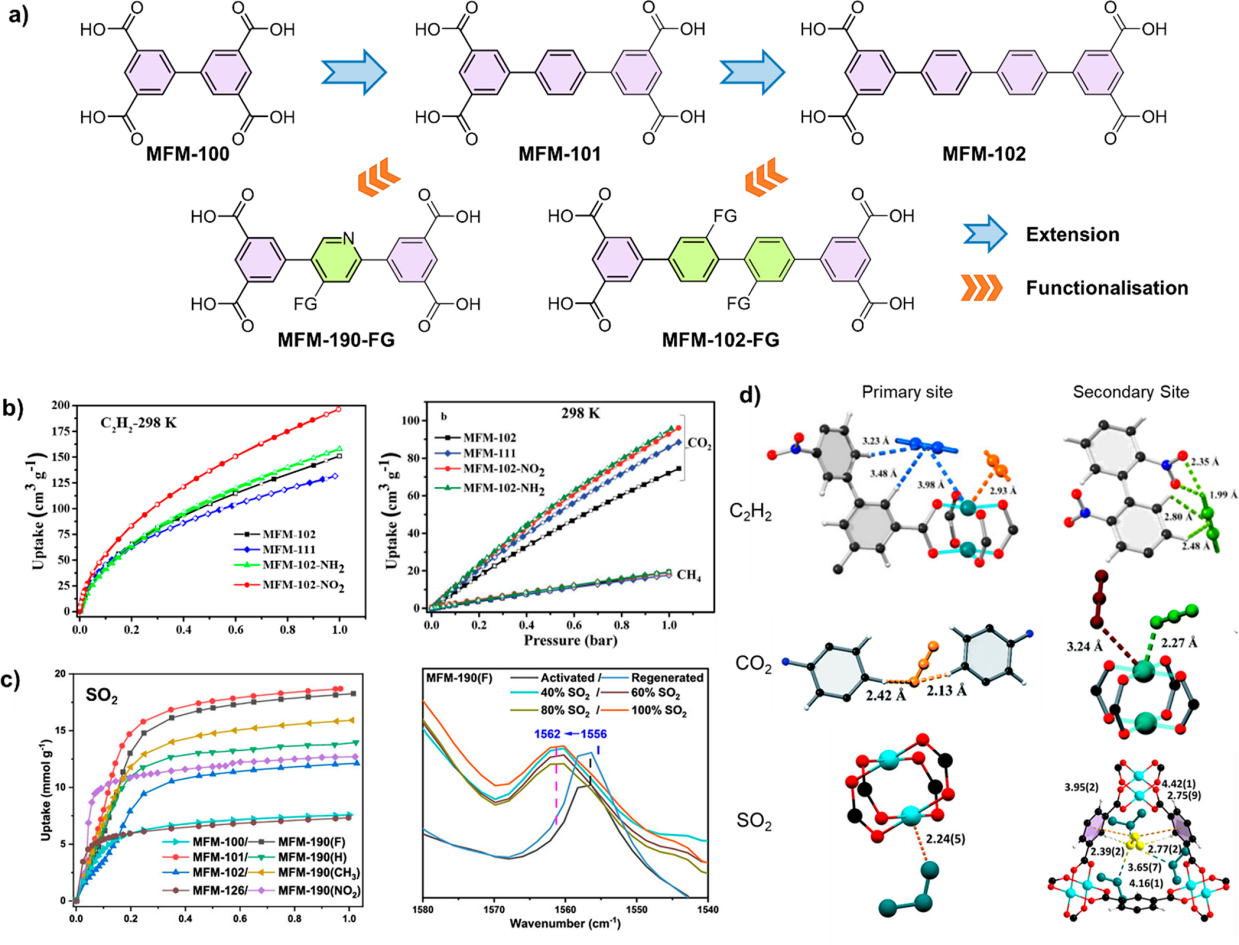
**a**) View of ligand functionalization (FG = functional group) in MOF linkers. **b**) C_2_H_2_ and CO_2_ adsorption in MFM-102-NO_2_ and related MOFs. **c**) SO_2_ adsorption in MFM-190-F and related MOFs. **d**) Views of the binding sites in functionalized MOFs. Reproduced with permission from ref (3), (36), and (37). Copyright 2022 and 2018 American Chemical Society; copyright 2020 Royal Society of Chemistry, respectively.

Several studies have demonstrated the effectiveness of ligand functionalization in enhancing adsorption performance. MFM-100/-101/-102 with a general formula of [Cu_2_L] (L^4–^ = di/tri/tetra-phenyl tetracarboxylates) are isoreticular MOFs composed of [Cu_2_(O_2_CR)_4_] paddlewheels bridged by tetracarboxylate linkers to afford *nbo*-type frameworks^35^ (Figure 3a). The impact of nitro, amine, and alkane functionalization in MFM-102 has been systematically studied for the adsorption of C_2_H_2_ and CO_2_.^36,37^ In both cases, MFM-102-NO_2_ shows the best performance, with an observed 28% increase in C_2_H_2_ (8.6 mmol g^–1^) and 36% increase in CO_2_ (8.2 mmol g^–1^) uptakes compared with those for the unfunctionalized material at 298 K and 1 bar. This is despite an overall reduction of the BET surface area by 15% upon the introduction of nitro groups (Figure 3b).

C_2_H_2_ features significant electron density in π-orbitals around its triple-bond axis, and is considered an electron-rich region. The interaction between C_2_H_2_ and the framework is thus affected greatly by the interaction of the π-electrons with open Cu(II) sites. The nitro group in MFM-102-NO_2_ provides further hydrogen bonding sites for the terminal H-centers of adsorbed C_2_H_2_ molecules. For CO_2_, the reverse order and a larger difference in electronegativity (+0.89 in CO_2_ vs −0.4 in C_2_H_2_) enable interaction with the electrophilic part of the framework. Thus, the main interaction shifts to the hydrogen bonding H_aromatic_···O=C=O with increased C–H acidity due to the presence of the electron-withdrawing nitro group on the benzene ring. The end-on coordination bond is also observed between CO_2_ and open Cu(II) sites.^36,37^

Interestingly, for gas molecules with more polar features, e.g., SO_2_, the observed host–guest interaction is distinct, and a good example is derived from MFM-101 incorporating pyridyl groups and other functional groups. The isostructural MFM-190-F exhibits a substantially higher adsorption of SO_2_ (18.3 mmol g^–1^ at 298 K 1 bar) compared with that of its analogues MFM-190-NO_2_, MFM-190-CH_3_, and MFM-190 (12.7, 15.9, and 14.0 mmol g^–1^, respectively) (Figure 3c).^3^ The most favorable binding site for SO_2_ is the open Cu(II) site while the −F group adjusts the acidity of the Cu(II) sites for enhanced interactions with the adsorbed SO_2_ molecules (Figure 3d). Additional interactions between SO_2_ and the phenyl ring are supplemented by blue shifts from 1556 to 1562 cm^–1^ of the phenyl ring distortion bands as observed by FTIR spectroscopy. Also, a higher value for *Q*_st_ is observed for MFM-190-F in comparison to the unfunctionalized analogue (35 and 29 kJ mol^–1^, respectively).

Electron-donating groups, such as amide and alkyne moieties, are also commonly selected for material functionalization. A tetra-amide functionalized material, MFM-188, exhibits exceptionally high CO_2_ (5.4 mmol g^–1^ at 298 K and 1 bar) and C_2_H_2_ (10.3 mmol g^–1^ at 295 K and 1 bar) uptakes with only moderate porosity (2568 m^2^ g^–1^).^38^ By changing the functionality from an amide to an alkyne group, the obtained framework MFM-127 demonstrates excellent C_2_H_2_ selectivity (C_2_H_2_/CO_2_ 3.7, C_2_H_2_/CH_4_ 21.2) in comparison to MFM-126 that is functionalized with amide groups.^39^ Structural studies confirm that the alkyne function is less favored than amide groups for CO_2_ binding due to the reduced ability to form hydrogen bonds in the former. Yet the highly confined pores of MFM-127 provide over half of the adsorbed C_2_H_2_ molecules with extensive hydrogen bonding and electrostatic interactions.

These examples illustrate the power of ligand functionalization to improve the adsorption and separation performance of MOFs.^40−42^ It is worth noting that the outcome of ligand functionalization may not always adhere to predictions. For example, the amide group in MFM-136 does not appear to bind directly to CO_2_ molecules as confirmed by NPD and inelastic neutron scattering (INS) experiments, even though the MOF shows very high adsorption of CO_2_ (12.6 mmol g^–1^ at 298 K and 20 bar).^43^ Thus, significant further effort is required to (i) deconvolute the impact of ligand functionalization from other factors that would contribute to the gas adsorption in MOFs, such as pore geometry, size, and open metal sites and (ii) reveal the indirect effects of ligand functionalization on overall gas adsorption.

### 2.2 Variation of Metal Centers

Metal centers are another fundamental component of MOFs and can provide direct and indirect binding sites to guest molecules. In systems where direct interactions are observed, open metal sites that can be generated by the removal of bound solvent molecules (e.g., water) often serve as the strongest binding sites.^44^ For example, activation of MFM-170·H_2_O, [Cu_2_(L)(H_2_O)], H_4_L = 4′,4‴-(pyridine-3,5-diyl)bis([1,1′-biphenyl]-3,5-dicarboxylic acid), affords one open Cu(II) site in each [Cu_2_(O_2_CR)_4_] paddlewheel within the framework.^2^ Coordinative binding of SO_2_ to the resultant open Cu(II) site in an end-on manner [O_SO_2__···Cu = 2.28(10) Å] is observed, resulting in an exceptional adsorption of SO_2_ (17.5 mmol g^–1^ at 298 K and 1.0 bar). A notable reduction of SO_2_ adsorption by ∼25% is observed upon blocking of this open Cu(II) site by water molecules, confirming their critical role.

**Figure 4:**
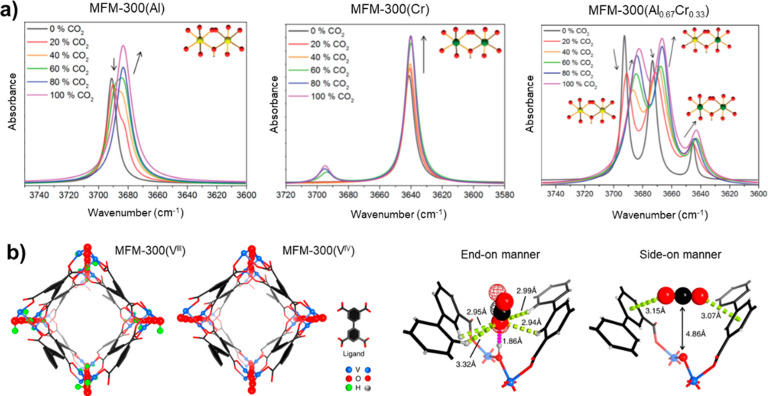
**a**) Views of μ_2_-OH stretching bands of MFM-300(Al, Cr, Al_0.67_Cr_0.33_) with increasing CO_2_ loading. **b**) Structures and binding of CO_2_ in MFM-300(V^III^) and MFM-300(V^IV^). Reproduced with permission from refs (1) and (46). Copyright 2017, 2021 Royal Society of Chemistry and Springer Nature, respectively.

In terms of indirect interactions, the elemental composition and oxidation state of metal centers can significantly influence the observed host–guest interactions and hence gas adsorption in MOFs.^45^ Constructing heterometallic MOFs is another promising approach to achieve fine-tuning of MOF properties. MFM-300(M) is a series of isostructural MOFs assembled from *cis*-[MO_4_(OH)_2_]_*n*_ chains linked by μ_2_-OH groups and bridged by 3,3′,5,5′-biphenyltetracarboxylate ligands (BPTC^4–^). MFM-300(Al_0.67_Cr_0.33_) demonstrates an enhancement in both SO_2_ uptake (8.1 to 8.6 mmol g^–1^ at 273 K and 1 bar) and SO_2_/CO_2_ selectivity compared with MFM-300(Al) as measured by ideal adsorbed solution theory (IAST).^46^ Using *in situ* synchrotron micro-IR spectroscopy, we observed the ν(OH) stretching modes in MFM-300(Al) and MFM-300(Cr) at 3690 and 3640 cm^–1^, respectively. In MFM-300(Al_0.67_Cr_0.33_), three distinct stretching modes are observed for Al–O(H)–Al, Al–O(H)–Cr, and Cr–O(H)–Cr at 3692, 3672, and 3644 cm^–1^, respectively, demonstrating the increase in μ_2_-OH acidity on Cr(III) doping (Figure 4a). The impact of metal centers on μ_2_-OH acidity is also reflected in the variation of the M–O bond length from 1.93(1) to 1.87(1) Å, leading to a shorter distance at primary binding sites, O_bridge_···O_SO_2__ = 3.16(1) Å compared with 3.20 (1) Å in the pristine Al-based framework MFM-300(Al).^47^

Variation in the oxidation state of metal centers is generally accompanied by changes in host–guest interactions.^1,48^ The postsynthetic oxidation of MFM-300(V^III^) [V^III^_2_(OH)_2_(L)] (L^4–^ = BPTC^4–^) to MFM-300(V^IV^) [V^IV^_2_O_2_(L)] results in an increase in the oxidation state at the V center with concomitant deprotonation of the bridging hydroxyl group to afford bridging oxy groups.^1^ The orientation of adsorbed CO_2_ molecules at the primary binding site in both frameworks is distinct: in MFM-300(V^III^), CO_2_ binds to the −OH group in an end-on manner, while in MFM-300(V^IV^), CO_2_ binds side-on to the bridging oxy group and is anchored between two phenyl rings. The variation in the host–guest interaction results in a higher *Q*_st_ value in MFM-300(V^III^) compared with MFM-300(V^IV^) (28 and 24 kJ mol^–1^, respectively), in line with the higher uptake (6.0 and 3.5 mmol g^–1^, respectively, at 298 K and 1 bar) (Figure 4b).

It should be noted that high densities of open metal sites are sometimes accompanied by a decrease in structural robustness, typically toward reactive molecules (e.g., H_2_O, SO_2_, NH_3_), and strategies to address such trade-offs need to be developed. Also, further control of the oxidation state of redox-active MOFs is required to achieve fine-tuning of the electronic properties of MOFs and to determine its impact on gas adsorption, particularly with reactive gas molecules.

### 2.3 Generation of Defects

The introduction of defects within materials can improve both catalytic and adsorption properties by generating reactive sites and domains to enhance host–guest interactions.^49,50^ Although several studies have demonstrated the benefits of defects in MOFs for gas adsorption,^51−53^ identifying and characterizing these defects structurally remains a significant challenge.^54−59^

**Figure 5:**
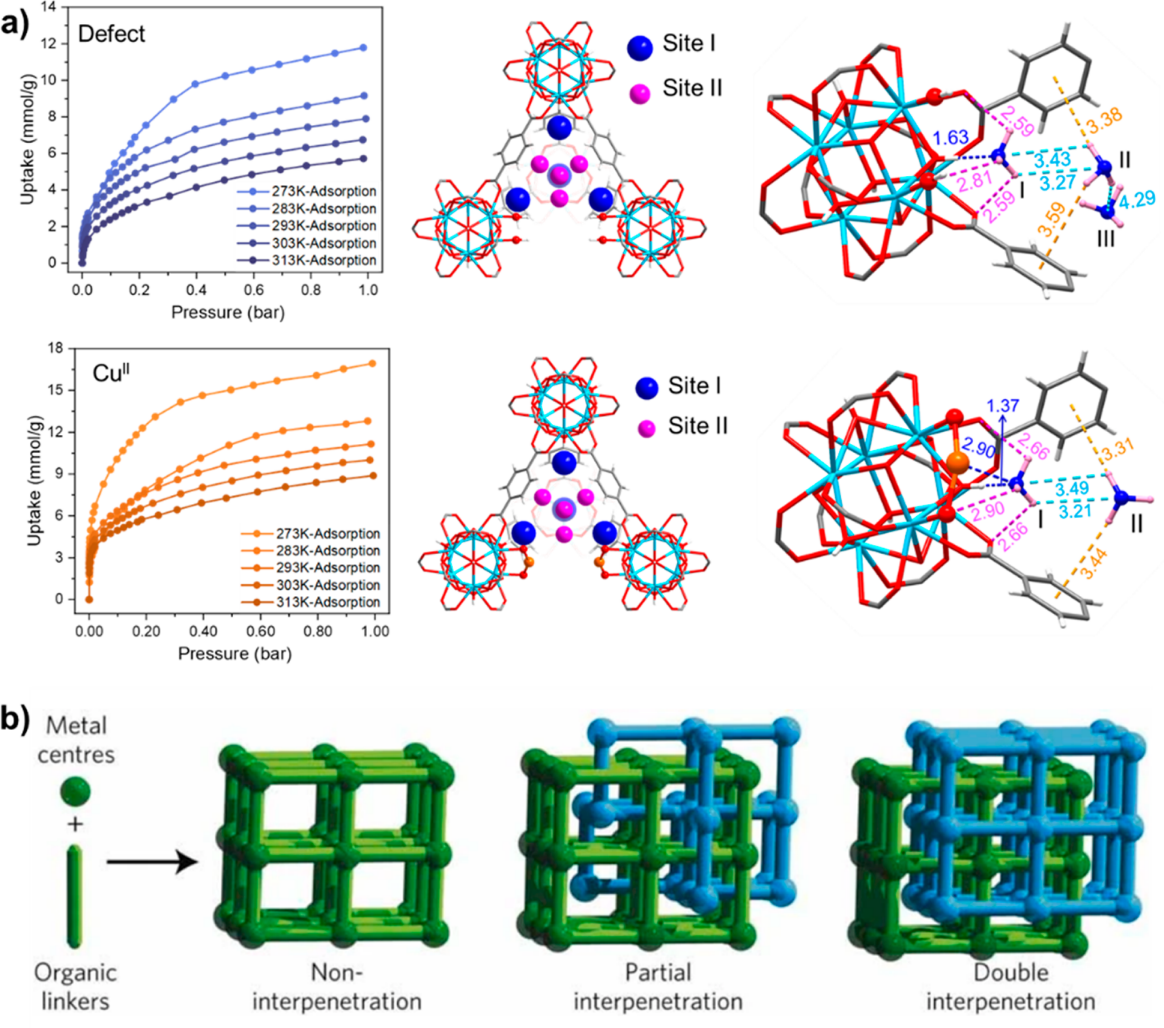
**a**) Adsorption and binding sites of NH_3_ in UiO-66-defect and UiO-66-Cu^II^. **b**) Illustration of the partially interpenetrated structure of MFM-202. Reproduced with permission from refs (66) and (67). Copyright 2022, 2012 American Chemical Society and Springer Nature, respectively.

Defects generated at metal cluster nodes via missing linkers can generate an increase in overall pore volume and give additional active sites either to interact with the guest molecules directly or to immobilize further metal centers subsequently.^60−62^ The generation of defects in UiO-66 materials using formic acid as a modulator has uncovered new possibilities for this ultra-stable material.^63,64^ With missing ligands to afford cluster defects, defective UiO-66 exhibits almost double the iodine uptake (2.25 g g^–1^) compared with that of the pristine material (1.17 g g^–1^).^65^ Atomically dispersed Cu(I) and Cu(II) sites can be introduced at defect sites by postsynthetic modification of UiO-66-defect to give UiO-66-Cu^I^ and UiO-66-Cu^II^ (Figure 5a).^66^ Compared with pristine UiO-66, the NH_3_ uptake at 273 K and 1 bar doubled in the UiO-66-defect and nearly tripled in UiO-66-Cu^II^ even though these materials exhibit similar BET surface areas (1111 and 1135 m^2^ g^–1^, respectively). NPD was used to study the host–guest interactions. In NH_3_@UiO-66-defect, the free hydroxyl groups generated at the defect site gave strong hydrogen bonding to NH_3_ at the primary binding site, 1.63(8)–1.96(1) Å. In UiO-66-Cu^II^, where the Cu(II) site is bound to the hydroxyl group, the primary binding site is jointly anchored by the Cu(II) site [Cu^II^···NH_3_ = 2.90(8)–3.00(6) Å) and adjacent hydroxyl groups through hydrogen bonding. Consistent with this, a higher value for *Q*_st_ was observed for UiO-66-Cu^II^ compared to UiO-66-defect at low surface coverage (55 and 35 kJ mol^–1^, respectively).

Framework-wide defects can also be generated during synthesis. For example, in MFM-202 (NOTT-202) [(Me_2_NH_2_)_1.75_[In(L)]_1.75_(DMF)_12_(H_2_O)_10_, H_4_L = biphenyl-3,3′,5,5′-tetra-(phenyl-4-carboxylic acid)], the interpenetrated framework consists of crystallographically independent nets A (occupancy 1.0) and B (occupancy 0.75), where net B is partially occupied and comprises two disordered, equally occupied nets B1 and B2 (Figure 5b).^67^ Connection between B1 and B2 nets is not possible due to steric and conformational effects, resulting in slit-shaped defects throughout the structure. This enables an unprecedented three-step CO_2_ adsorption isotherm at 195 K from the sequential filling of guest molecules into pores with different dimensions. Crystallographic studies show a reversible peak shift, confirming the presence of framework flexibility. SO_2_ adsorption in MFM-202 also results in a stepped isotherm but with an irreversible phase transition in the diffraction study due to the formation of strong host–guest interactions that stabilized the new phase which cannot be obtained from solvothermal synthesis.^68^ Subsequently, through an autocatenation process, the occupancy of one sublattice in interpenetrated MOFs was gradually modified to afford more diverse defect structures.^69^

These examples demonstrate the great potential of structural defects in tuning and promoting gas adsorption and framework flexibility in MOFs. Compared with ligand functionalization, the manipulation of defects does not necessarily induce notable changes in the porosity of the materials and thus affords the unique advantage to optimize the gas capture at low pressure owing to the enhanced host–guest interactions while maintaining (or even increasing) the total adsorption capacity at high pressure and full saturation. Further efforts in synthesis and characterization are required to better control the generation and understanding of the chemistry of structural defects in MOFs for gas adsorption and, more interestingly, for catalysis.

### 2.4 Addition of Single Atom Sites

Rational incorporation of single metal sites into MOF structures can enhance their adsorption and catalytic performance.^70,71^ These active metal sites can be introduced by postsynthetic modification and distributed evenly and at defined positions throughout the porous structure. The resulting stronger host–guest interactions thus generated by these single active sites can contribute to higher adsorbate concentrations and, in some cases, afford different binding configurations with a lower activation barrier. Single atom sites in the framework can adopt various forms based on the synthetic routes, including ligand-bound metal complexes, mixed metal nodes, and pore-trapped metal ions, atoms, and clusters.^72,73^

**Figure 6:**
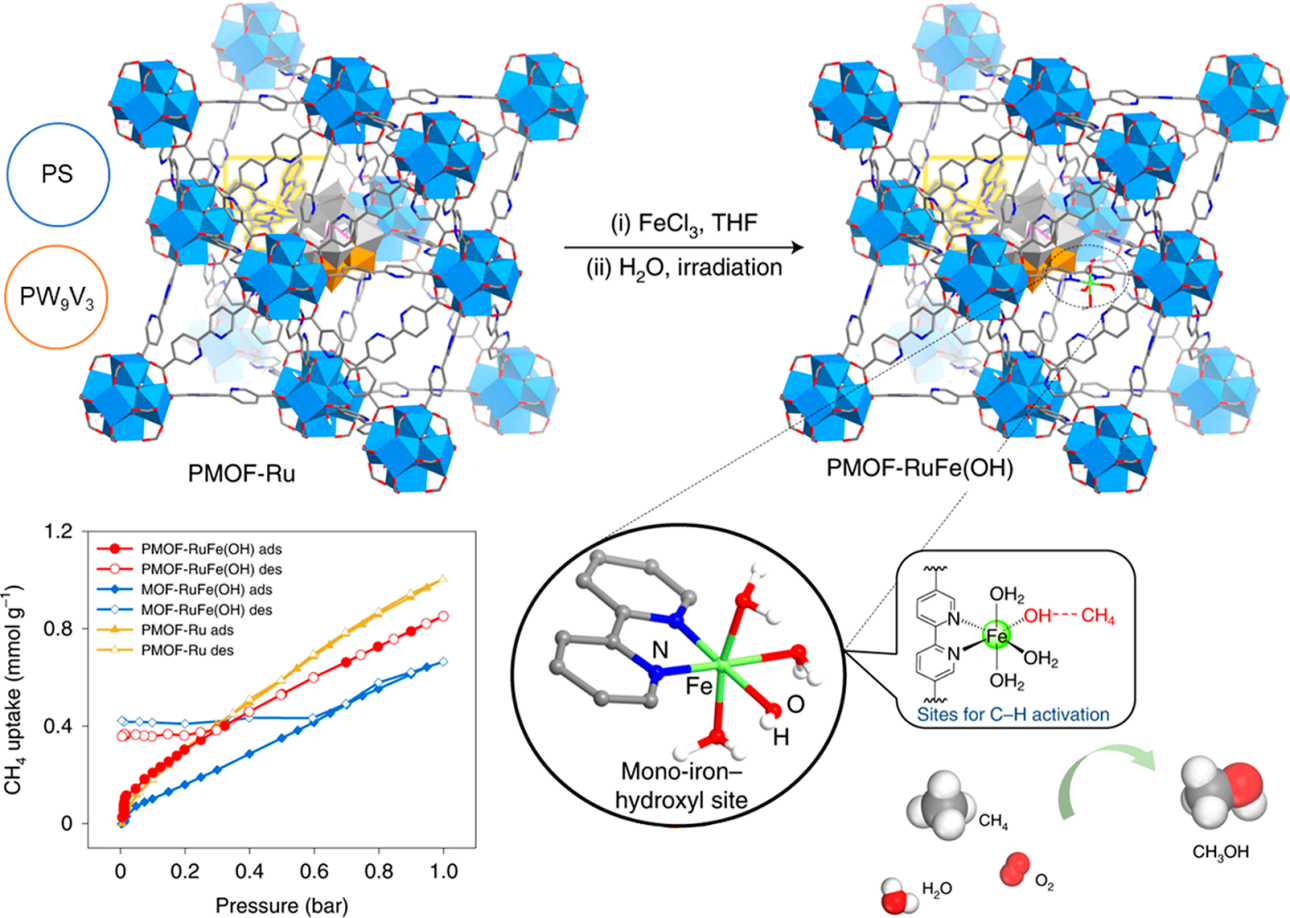
Insertion of a single iron-hydroxyl site, CH_4_ adsorption isotherms, and CH_4_ catalysis by PMOF-RuFe(OH). Reproduced with permission from ref (4). Copyright 2022 Springer Nature.

Methane adsorption and conversion in MOFs can be tuned through the addition of a monoiron hydroxyl site inspired by biosystems^4^ (Figure 6). PMOF-Ru, a functionalized UiO-67 material, can be obtained via one-pot synthesis of the parent MOF but with the addition of photosensitizer [Ru^II^(bpy)_2_(bpydc)] (bpy = 2,2′-bipyridine, H_2_bpydc = 2,2′-bipyridine-5,5′-dicarboxylic acid) and redox-active polyvanadotungstate [PW_9_V_3_O_40_]^6–^. Postsynthetic metalation with FeCl_3_·6H_2_O yields PMOF-RuFe(Cl), and pretreatment in water with light produces the active material, PMOF-RuFe(OH). CH_4_ isotherms of PMOF-RuFe(OH) exhibit an initial steep rise and high residue postdesorption (0.5 CH_4_/Fe), which is not observed in PMOF-Ru (without Fe sites). INS combined with density functional theory (DFT) simulations illustrates the interaction between [(bpy)Fe(OH)(H_2_O)_3_]^2+^ and CH_4_ molecules, primarily based on hydrogen bonding (Fe–OH···CH_4_ = 2.39 Å). Functional organic linkers, such as porphyrins, can also provide coordination sites for metal ions. In MOF-525, [Zr_6_O_4_(OH)_4_(TCPP-H_2_)_3_] [TCPP = 4,4′,4′′,4‴-(porphyrin-5,10,15,20-tetrayl) tetrabenzoate], coordinatively unsaturated Co(II) sites are bound to the porphyrin to produce MOF-525-Co.^74^ A nearly 30% increase in CO_2_ uptake is observed in MOF-525-Co compared with that in the pristine MOF, along with a lower activation energy barrier, resulting in a 3.13-fold improvement in the rate of reduction of CO_2_ to CO.

These examples highlight the potential of incorporating single metal sites into MOF structures to enhance the adsorption and catalytic performance. This research is still in its infancy, and there is plenty of opportunity to translate bioinspired systems with single or dual metal sites into MOF structures to produce artificial host systems to uncover unprecedented properties for adsorption, activation, and catalysis of small molecules.

### 2.5 Design and Control of Pore Structure

**Figure 7:**
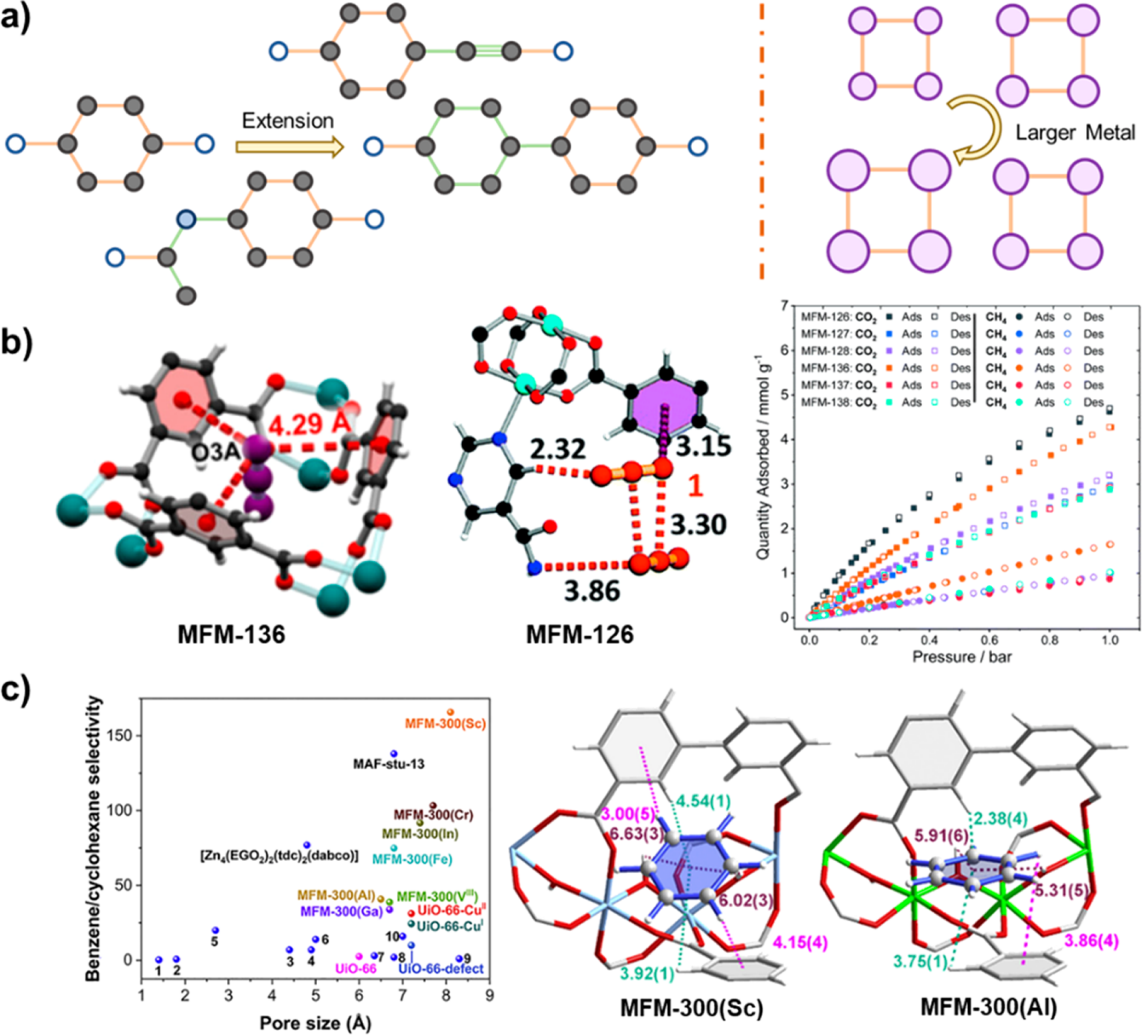
**a**) Scheme of common strategies for adjusting the pore size through ligand extension or metal alteration. **b**) Views of primary binding sites and adsorption of CO_2_ in MFM-136 and MFM-126, where a smaller pore is presented in the latter. Reproduced with permission from refs (39) and^43^. Copyright 2019, 2016 Royal Society of Chemistry and American Chemical Society, respectively. **c**) Separation performance of benzene vs cyclohexane in MFM-300(M) with subangstrom changes in pore size; views of the binding of benzene in MFM-300(Sc) and MFM-300(Al), which has largest and smallest pores, respectively, in the series. Reproduced with permission from ref (76). Copyright 2023 Elsevier.

Confinement of gas molecules within the pores through control of the pore size and shape can further improve the host–guest interactions. Reticular chemistry allows the elongation or shortening of ligands (typically via phenyl rings and acetyl groups) without altering the framework topology (Figure 7a), thereby enabling the regulation of pore dimensions by approximately 2–4 Å for each step.^13,31,35^ For more precise regulation, introducing additional moieties such as amide and alkyne groups into the skeleton of the linker ligands can be employed, further enhancing the ability to fine-tune the pore dimensions (Figure 7b).^39^ In light of the finding of MFM-136,^43^ where the adsorption performance of CO_2_ is not driven by direct interaction with the amide group but rather by a combination of geometry, pore size, and functionality, MFM-126/-127/-128/-136/-137/-138 were synthesized.^39^ These variants, which possess shortened or elongated ligands, maintain a consistent *eea* topology and demonstrate an adjustable pore size. MFM-126, which exhibits the smallest pore size (12.3 × 15.4 Å^2^ vs 16.2 × 24.9 Å^2^ in MFM-136), demonstrates the highest CO_2_ uptake of 7.00 mmol g^–1^ at 1 bar and a comparatively low CH_4_ uptake of 1.50 mmol g^–1^ in this series. The enhanced CO_2_ adsorption performance is consistent with the observed increased value for *Q*_st_. This stronger interaction is associated with the narrower pore with the primary CO_2_ binding site demonstrating cooperative binding to the amide group, enabled by reduced steric effects (Figure 7b).

By utilizing the relatively small differences in the size of metal ions that constitute the MOF, the pore size can be adjusted in steps of 0.1 Å.^75^ By varying the metal center in MFM-300(M) (M = Al, Sc, V, Cr, Fe, Ga, and In), the pore size can be tuned precisely from 6.3 to 8.1 Å and can be used for the selective separation of benzene and cyclohexane^76^ and of *ortho*-, *meta*-, and *para*-xylenes (Figure 7c).^77^ Despite the similarities between aromatic compounds, xylenes and benzene exhibit different affinities toward MFM-300(M) when different metals are used. MFM-300(Sc), with the largest pore diameter of 8.1 Å, displays the highest uptake of benzene of 3.01 mmol g^–1^ at 1.2 mbar and 298 K with the highest value *Q*_st_ within this series. Due to the absence of π···π interaction with cyclohexane, the uptake of cyclohexane in MFM-300(M) does not vary significantly (1.27–2.46 mmol g^–1^ at 298 K and 1.3 mbar). Thus, the separation performance of benzene vs cyclohexane shows a high correlation with pore size and benzene uptake, where a large pore size within the range of 6.3 to 8.1 Å is beneficial for the separation.^76^ In the case of xylene separation, the highest isothermal uptake of all three isomers is achieved by MFM-300(Al), where the narrowest pore fosters strong interactions with the xylene isomers through π···π interactions at distances of 3.5–3.7 Å. However, MFM-300(Al) exhibits the poorest separation performance due to the restricted diffusion of xylene molecules. With a larger pore, differences in π···π interaction between the framework and the xylene isomers become apparent, and in MFM-300(In), the refinement in differences between framework–substrate and substrate–substrate interactions for *para*- and *meta*-xylene enables their excellent separation. Furthermore, a two-column system comprising MFM-300(In) and MFM-300(V) used in series leads to the effective separation of all three xylene isomers.^77^

The physical similarities between target guest molecules lead to challenges in the ability to separate them efficiently. The control of pore size and shape is of critical importance and has been proven particularly effective for the adsorption of C6–C8 hydrocarbons as above, where the dimensions of the substrates are matched and tuned closely to the available pore size and shape. Thus, the separation of molecules with only minor differences in kinetic radii can be achieved via the precise and detailed regulation of host pores.

## 3 Conclusions and Outlook

In this Account, we have presented a summary of the binding interactions between MOFs and various gas molecules derived from a structure-based MOF data set in the CSD. We clarify the relationship between uptake and surface area, and for H_2_ and CH_4_, the surface area primarily dictates uptake, especially at saturation. For more polar CO_2_, C_2_H_2_, and SO_2_, where their structures enable stronger, direct binding to the host via multiple hydrogen or coordinative bonds, the host–guest interaction substantially impacts the adsorption performance, especially at low pressures. To illustrate its important role, we have reviewed the available strategies, including ligand functionalization, variation of metal centers, generation of defects, addition of a single atom site, and design and control of pore structure, all of which can be employed individually or in combination to modify host–guest interactions and thus optimize adsorption and separation performances. Structural analysis by X-ray or neutron diffraction and scattering experiments gives important insights into understanding how individual or families of materials operate or not as the case may be. Although the structural determination of appropriate substrate-bound MOFs remains limited, the proliferation of high-quality X-ray diffraction techniques, more accessible synchrotron and neutron facilities, and the emergence of new technologies such as electron diffraction and advanced spectroscopy will aid future investigation. To date, research on guest-loaded MOFs has focused primarily on examining and determining interactions with single-component gases/substrates. This approach offers limited insights into competitive adsorption, especially for systems tailored for gas separation. New developments in analytical analysis and associated techniques that can reveal data on the competition of adsorption are highly desirable.

Defects and single metal sites have been highlighted in order to promote catalysis. However, their significance in adsorption applications can be overlooked. Additionally, the precise control of density and distribution of defects and single metal sites is at the forefront of investigations, and the manipulation of active sites and examining the local interactions between active and defect sites and guest molecules are highly timely areas. Likewise, the roles of functional groups and their relationships and effects on adsorption or separation performance are active areas of investigation. For similarly functionalized MOFs, the influence of the functional group can vary and occasionally yield unexpected outcomes. To better understand the role of functionality, the development of an integrated toolbox of experimental and computational techniques is necessary.

We anticipate that this Account will stimulate further research interest in the structural characterization, rational design, and mechanistic studies of host–guest interactions in MOFs to promote the development of advanced sorbent materials for challenging gas adsorption, separation, and catalysis.

## Supporting Information Available

Protocol to build the database and additional analysis of the host–guest interaction for entries in the database (PDF)
