# Supplementary material for: Analysis and Refinement of Host–Guest Interactions in Metal–Organic Frameworks
Source: Acc Chem Res. 2023 Aug 30;56(19):2569–81. doi: 10.1021/acs.accounts.3c00243 (PMC10552526; doi:10.1021/acs.accounts.3c00243)
Supplement: Supplementary file 1 — ar3c00243_si_001.pdf [file ar3c00243_si_001.pdf]

Supporting Information

Analysis and Refinement of Host-Guest Interactions in Metal-Organic Frameworks

Yinlin Chen,[1#] Wanpeng Lu,[1#] Martin Schröder[1]* and Sihai Yang[1,2]*

1. Department of Chemistry, University of Manchester, Manchester, M13 9PL, UK

*M.Schroder@manchester.ac.uk; *Sihai.Yang@manchester.ac.uk

2. College of Chemistry and Molecular Engineering, Beijing National Laboratory for Molecular Sciences, Peking University, Beijing 100871, China. *Sihai.Yang@pku.edu.cn

# These authors contributed equally to this work.

1. Construction of the MOF dataset in this Account:

According to the International Union of Pure and Applied Chemistry (IUPAC) definition, a metal-organic framework (MOF) is a coordination network with organic ligands containing potential voids. Three key features can be extracted from this definition: coordination bonds, 2D or 3D expansion, and the presence of potential voids. Based on these characteristics, an algorithm was developed to filter 3D-structured MOFs from the Cambridge Structural Database (CSD).

The algorithm initially identified possible coordination bonds by assessing the distance between metal and non-metal atoms. As organic building blocks are essential for the construction of MOFs and always contain carbon, inorganic ligands with carbon, such as cyanide, thiocyanide, or carbonate, were excluded from the list. Next, the maximum degrees of extension are tested, taking into account of the interpenetration. Finally, after removal of neutral solvents and monodentate ligands within the pore and mitigating internal disorder, the pore volume was assessed using a probe radius of 1.2 Å.

In v5.43 of the CSD, which contains a total of 1.18 million structures, 686,000 entries incorporate metals, and 200,000 entries exhibit coordination bonds to organic ligands. The structural skeleton was generated by removing monodentate ligands and guest molecules, then subjected to a test for maximum expansion dimensions. Due to the prevalence of adsorption studies on 3D-MOFs, only these structures were included for further investigation. Among all coordination complexes with organic linkers, 46% are isolated structures (zero-dimension); 33% exhibit expansion in certain dimensions but not all three, while only 40,839 structures can fully expand in three dimensions. In most cases, neutral guest molecules within the pores can be readily removed through activation or solvent exchange, while the neutral monotopic linkers, typically organic solvents, can be eliminated during the activation process, affording potential voids. Therefore, potential voids were examined in the 3D-MOF candidates based on a probe radius of 1.2 Å. A total of 33,931 entries in the database possess potential voids and were collected for the MOF dataset in this Account.

Guest species were extracted from the structures, while monodentate ligands with a focus on neutral (uncharged) systems for inclusion in the list. It was observed that some cations and anions appear in the list, balancing the charges of the framework. Aside from these ions, the most common species found within the pores were those that typically function as solvents, including water, dimethyl formamide, methanol, and acetonitrile. Additionally, some gas molecules intended for adsorption studies are present in the list, with carbon dioxide (CO_2) being the most prevalent with 1,972 entries. The distribution of

guest species and monotopic compounds are listed in the **Table S1**. For the neutral species, their distribution is described in the word cloud (**Figure S1**).

The host-guest interaction and adsorption performance of a given MOF were extracted from the structural analysis and corresponding literature, respectively. To discuss the relationship more generally, the conditions of adsorption performance were compared at the most commonly reported conditions, which are 298 K, 1 bar for C_2H_2 and CO_2; 273 K, 1 bar for SO_2; and 77 K, 1 bar for H_2. In **Tables S2–6**, material name, metal, BET surface area, possible active site, main interaction, primary interaction with its distance, secondary interaction with its distance (only for CO_2) and the reference are listed for selected examples. The type of possible active site includes open metal site (OMS in the Table), cation or anion, and functional group on the metal cluster or on the ligand. For the host-guest interaction, we firstly determined the type of the dominating interaction as hydrogen bond (denoted as H-Bond in the Table, see below), coordination bond (CN), electrostatic interaction to charged component (ES(charged)), electrostatic interaction (ES), π-system related interactions (p-π, π-π or H-π). To distinguish the host and guest components, the host is always in front of the dash and the guest after.

The relationships between the uptake and Q_{st} for H_2 (77 K, 1 bar), CO_2 (298 K, 1 bar) and C_2H_2 (298 K, 1 bar) for selected MOFs are shown in **Figure S2**.

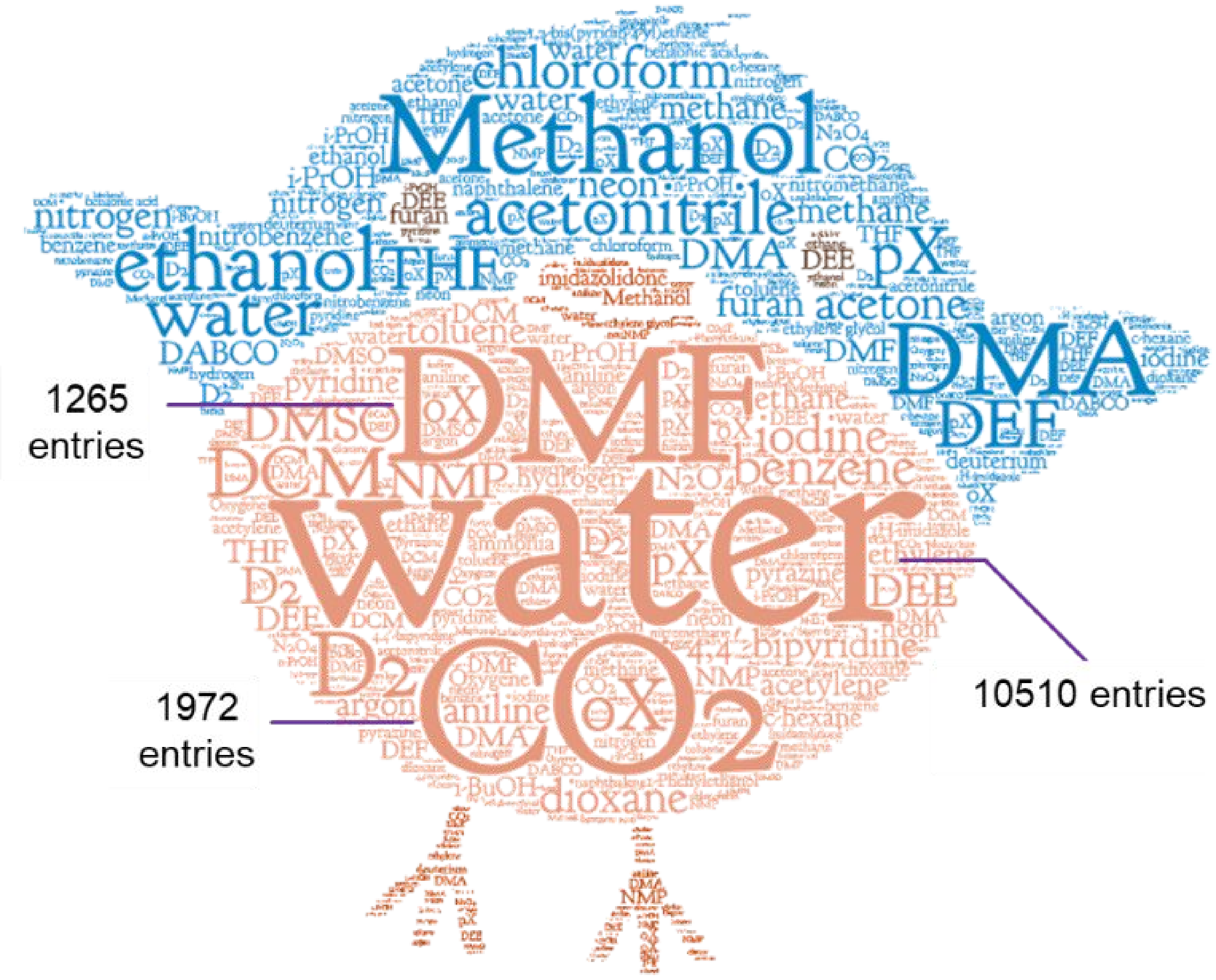

Figure S1 Word Cloud of neutral monodentate ligands and neutral guest molecules appearing in this MOF dataset.

Table S1 The distribution of all guest/monodentate ligands in the MOF dataset (solv = solvent)

Species	Common Name	Entries	Type	Species	Common Name	Entries	Type
H_2O	Water	10510	solv	NMe_4^+	Tetramethylammonium	91	cation
CO_2		1972	guest	C_6H_6	Benzene	88	solv
Me_2NCHO	Dimethylformamide	1265	solv	Br^-		87	anion
$NH_2Me_2^+$	Dimethylammonium	579	cation	$C(NH_2)_3^+$	Guanidinium	83	cation
ClO_4^-	Perchlorate	412	anion	$MeNH_3^+$	Methylammonium	82	cation
MeOH	Methanol	381	solv	OH^-		69	anion
NO_3^-	Nitrate	370	anion	$C_6H_5NO_2$	Nitrobenzene	69	solv
NH_4^+	Ammonium	269	cation	$EtNH_3^+$	Ethyl ammonium	61	cation
MeCN	Acetonitrile	250	solv	I_2		59	guest
Me_2NCMeO	Dimethylacetamide	247	solv	Me_2SO	Dimethyl sulfoxide	59	solv
Cl^-		240	anion	CH_2Cl_2	Dichloromethane	59	solv
EtOH	Ethanol	227	solv	Me_2CO	Acetone	55	solv
OH_3^+	Hydronium	191	cation	$N_2H_5^+$	Hydrazinium	54	cation
BF_4^-	Tetrafluoroborate	152	anion	CH_4	Methane	51	guest
C_6H_{12}	*c*-Hexane	136	guest	Et_2NCHO	Diethyl formamide	49	solv
K^+		133	cation	$C_4H_8O_2$	Dioxane	48	solv
Na^+		127	cation	H_2		45	guest
$CHCl_3$	Chloroform	125	solv	NEt_3H^+		41	cation
CF_3OSO^-	Triflinate	101	anion	C_2H_2	Acetylene	37	guest
PF_6^-	Hexafluorophosphate	94	anion	SO_2		11	guest

* Me = $–CH_3$, Et = $–CH_2CH_3$

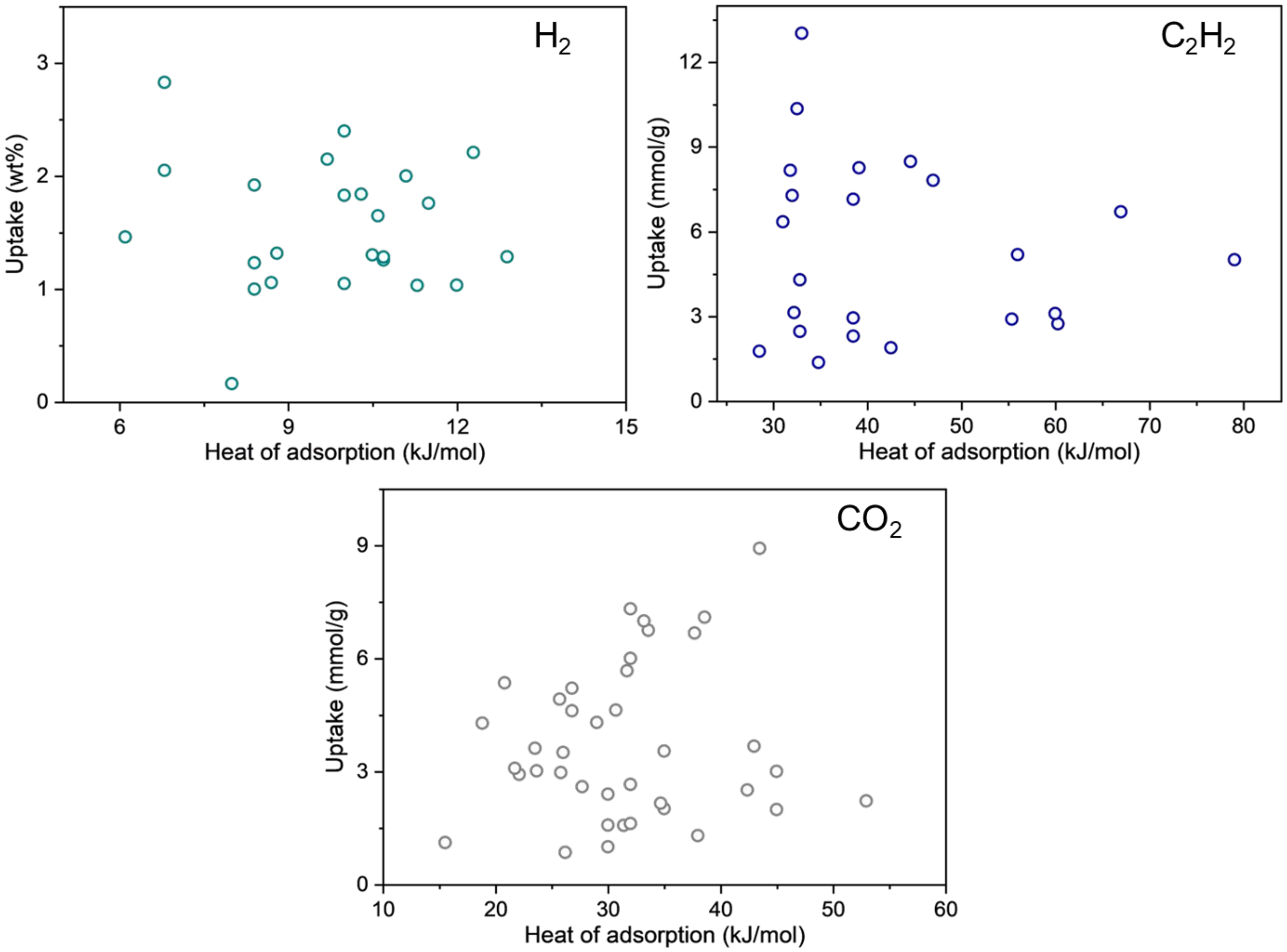

Figure S2 The relationship between the uptake and Q_{st} for H_2 (77 K, 1 bar), CO_2 (298 K, 1 bar) and C_2H_2 (298 K, 1 bar) for selected MOFs from host-guest structural information in the CSD database.

Table S2 The main interaction of CO_2-contained MOFs

Material	Metal	Active site	Main Interaction*	Primary interaction	Distance (Å)	Secondary interaction	Distance (Å)	Reference
MFM-300(In)	In	OH	H-Bond	OH−O	3.04			1
PCN-200-syn	Cu		H-Bond	CH−O	2.63/2.75			2
CaSDB	Ca		p-π	π−C	3.80/3.81			3
$Cu_2(glu)_2(bpy)$	Cu		H-Bond	CH−O	3.109			4
$Cu_2(glu)_2(bpe)$	Cu		H-Bond	CH−O	2.714	O−C	3.254	4
$Cu_2(glu)_2(bpymh)$	Cu		H-Bond	CH−O	2.648	O−C	3.233	5
Sc_2BDC_3	Sc		H-bond	CH−O	2.78−2.98			6
Zn(tp)(1,2,3-tz)	Zn		H-bond	CH−O	3.0−3.2	C−O	3.12	7
Zn(imPim)	Zn		H-bond	CH−O	3.0−3.2	p−π	3.5	8
SIFSIX-Cu-TPA	Cu	SiF_6	ES(charged)	SiF−C	2.971	CH−O	2.9	9
Cd-NP	Cd		ES(charged)	Cd−O	3.187			10
NKU-100	Ni	OMS	CN	Ni−O	2.104			11
PMOF-55	Zn		H-Bond	CH−O	3.22−3.30			12
NH_2-PMOF-55	Zn	NH_2	H-Bond	NH−O	3.32			12
FJU-40-NH_2	Zn	NH_2	H-Bond	CH−O	3.00−3.41			13
NH_2-MIL-53(Al)	Al	NH_2	H-Bond	N−O	2.339			14
$Zn_3(OH)_2(pzdc)(tz)$	Zn	OH	H-Bond	OH−O	2.55			15
$Zn_2(dobpdc)$	Zn	OMS	CN	Zn−O	2.528			16
PbSDB	Pd		p-π	π−C	3.55			17
Cu(INAIP)	Cu		ES	O−C	2.911	CH−O	2.52–3.05	18
Cu_2(S,S)-hismox	Cu	OMS	CN	Cu−O	3.0−3.4	CH−O	3.4	18
Cu–BTTri	Cu	OMS	CN	Cu−O	2.86			19
$CuI_2(py\text{-}pzpypz)_2(\mu\text{-}CN)_2$	Cu		H-Bond	CH−O	2.85	Cu−O	4.271	20

MFM-300(Ga)	Ga	OH	H-Bond	OH−O	1.883			21
FJU-14-BF_4-a	Cu		ES	O−C	3.031			22
$Zn_2(tdc)_2dabco$	Zn		ES	S−C	3.49			23
$Mg(H_2gal)$	Mg	OH	H-Bond	OH−O	2.16			24
SIFSIX-14-Cu-i	Cu	SiF_6	ES(charged)	SiF−C	2.57	CH−O	2.61	25
NKMOF-3-Ln	La		ES	O−C	3.05−3.07			26
α-$Zn_3(HCOO)_6$	Zn		H-Bond	CH−O	2.91−3.03			27
Co-DpyDtolP	Co		H-Bond	CH−O	4.2			28
SNU-9	Zn		H-Bond	CH−O	2.56			29
MAF-23	Zn		ES	N−C	2.84−3.04			30
MAF-2	Cu		H-Bond	Cu−O	3.78	CH−O	2.865	31
MFM-520	Zn		H-Bond	CH−O	3.2−3.5			32
PCP-N	Zn		ES	N−O	3.23	CH−O	3.50	33
NbOFFIVE-1-Ni	Ni		ES(charged)	NbF−C	3.05	CH−O	3.05	34
MFM-136	Cu		p-π	π−O	3.22	CH−O	2.33	35
$Zn_2(dobpdc)$	Zn	OMS	CN	Zn−O	2.528			36
MFM-601	Zr		ES	Zr−O	2.4			37
MFM-300(Al)	Al	OH	H-Bond	OH−O	2.335	O−C	2.00	38
MFM-188a	Cu	OMS	CN	Cu−O	2.34			39
MFM-300(V)	V(III)	OH	H-Bond	OH−O	1.863	CH−O	2.984	40
MFM-300(V)	V(IV)		p-π	O−π	2.741			40
UTSA-16	Co		H-Bond	OH−O	2.971			41
MAF-49	Zn		H-Bond	CH−O	3.33			42
Zn-DPA	Zn		ES(charged)	Zn−O	3.17			43
ZIF-7	Zn		H-Bond	CH−O	1.676/2.2			44
MOF-74	Cu	OMS	CN	Cu−O	2.86			45

MOF-74	Mn	OMS	CN	Mn−O	2.51			45
MOF-74	Zn	OMS	CN	Zn−O	2.43			45
MOF-74	Mg	OMS	CN	Mg−O	2.23			45
MOF-74	Ni	OMS	CN	Ni−O	2.29			45
MOF-74	Fe	OMS	CN	Fe−O	2.29			45
MOF-74	Co	OMS	CN	Co−O	2.29			45
$Fe_2(azpy)_4(NCS)_4$	Fe	BH	H-Bond	BH−C	2.9	CH−O	2.76	46
MFM-305	Al	OH	H-Bond	OH–O	2.54	CH−O	2.4	47
MFM-305-Me	Al	Cl	ES(charged)	Cl−C	3.78	CH−O	2.54	47
MFM-126	Cu		H-Bond	p−π	3.15	CH−O	2.32	48
MFM-127	Cu		ES	C−O	2.87−3.55	CH−O	2.27	48
MFM-300(Cr)	Cr	OH	H-Bond	OH−O	3.341	CH−O	3.223	49

Table S3 The reported adsorption performance of MOFs for CO_2

Material	Metal	BET (m^2/g)	Uptake (298, 1bar, mmol/g)	Q_{st} (kJ/mol)	Active site	Main Interaction	Primary interaction	Distance (Å)
MOF-74	Mg	1957	8.93	43.5	OMS	CN	Mg−O	2.23
NKU-100	Ni	698	3.67	43	OMS	CN	Ni−O	2.10
MOF-74	Ni	1574	7.10	38.6	OMS	CN	Ni−O	2.29
MOF-74	Co	1438	6.75	33.6	OMS	CN	Co−O	2.29
MOF-74	Fe	1536	7.00	33.2	OMS	CN	Fe−O	2.29
MOF-74	Mn	1797	5.672	31.7	OMS	CN	Mn−O	2.51
MOF-74	Zn	1277	4.606	26.8	OMS	CN	Zn−O	2.43
MOF-74	Cu	1515	2.92	22.1	OMS	CN	Cu−O	2.86
Cu–BTTri	Cu	1850	3.08	21.7	OMS	CN	Cu−O	2.86
MFM-188a	Cu	2568	5.35	20.8	OMS	CN	Cu−O	2.34
Zn_2(dobpdc)	Zn	3440			OMS	CN	Zn−O	2.53
Cu_2(S,S)-hismox	Cu	624	1.46		OMS	CN	Cu−O	3.00-3.40
MFM-127	Cu	1557	2.97	25.8		ES	C−O	2.87-3.55
$Zn_2(tdc)_2$dabco	Zn	1553	3.01	23.65		ES	S−C	3.49
FJU-14-BF4-a	Cu	324	4.28	18.8		ES	O−C	3.03
MFM-601	Zr	3644	1.10	15.5		ES	Zr−O	2.40
NbOFFIVE-1-Ni	Ni	280	2.21	53		ES(charged)	NbF−C	3.05
SIFSIX-14-Cu-i	Cu	612	6.68	37.7	SiF_6	ES(charged)	SiF−C	2.57
Zn-DPA	Zn	283	1.56	31.4		ES(charged)	Zn−O	3.17
MFM-305-Me	Al	256	2.39	30	Cl	ES(charged)	Cl−C	3.78
Cd-NP	Cd	305	2.59	27.7		ES(charged)	Cd−O	3.19
SIFSIX-Cu-TPA	Cu	1330	4.92	25.7	SiF_6	ES(charged)	SiF−C	2.97

PCN-200-syn	Cu	157.1	1.285	38		H-Bond	CH−O	2.63/2.75
MFM-520	Zn	313	2.01	35		H-Bond	CH−O	3.20-3.50
MFM-300(Ga)	Ga	392	2.15	34.7	OH	H-Bond	OH−O	1.88
NH_2-PMOF-55	Zn	453	1.61	32	NH_2	H-Bond	NH−O	3.32
MFM-300(V)	V	1892	6.00	32	OH	H-Bond	OH−O	1.86
UTSA-16	Co	628	7.32	32		H-Bond	OH−O	2.97
MFM-305	Al	779	2.65	32	OH	H-Bond	OH	2.54
MFM-126	Cu	1004	4.63	30.7		H-Bond	p−π	3.15
PMOF-55	Zn	371	1.57	30		H-bond	CH−O	3.22–3.30
MFM-300(Al)	Al	1370	4.30	29	OH	H-Bond	OH−O	2.34
MFM-300(Cr)	Cr	1360	5.21	26.8	OH	H-Bond	OH−O	3.34
MFM-300(In)	In	1071	3.61	23.5	OH	H-Bond	OH−O	3.04
Sc_2BDC_3	Sc	721	1.10 (303 K)	20		H-Bond	CH−O	2.78-2.98
Zn(tp)(1,2,3-tz)	Zn	210.8	1.67			H-Bond	CH−O	3.0-3.2
FJU-40-NH_2	Zn	659.3	2.017		NH_2	H-Bond	CH−O	3.00-3.41
CaSDB	Ca	145.1	0.989	30		p-π	π−C	3.80/3.81
MFM-300(V)	V(IV)	1565	3.50	26		p-π		2.74
MFM-136	Cu	1634		25.6		p-π	π−O	3.22

Table S4 The reported adsorption performance and the host-guest interaction of MOFs for H_2

Material	Metal	BET (m^2/g)	Uptake at 77 K, 1 bar (wt%)	Q_{st} (kJ/mol)	Active site	Main Interaction	Primary interaction	Distance (Å)	Reference
CPL-1	Cu	414	0.8			ES	O−H	3.222	50
$Cr_3[(Cr_4Cl)_3(BTT)_8]_2$	Cr	2030	2.4	10.0	OMS	CN	M−H	2.57	51
Cu-BTTri	Cu	1950	1.3	10.5	OMS	CN	M−H	2.73	52
MFM-132a	Cu	2466	2.83	6.8	OMS	CN	M−H	2.07	53
Cu^{I}-MFU-4l	Cu		0.34	32.0	OMS	CN	M−H	1.60	54
MFM-300(In)	In	1071	2.05	6.8		ES	OH−H	2.54	55
MOF-5	Zn	3800	1.50			ES	C−H	3.39	56
CPO-27–Mn	Mn	1369	1.92	8.4	OMS	CN	M−H	2.67	57
CPO-27–Cu	Cu	1369	1.46	6.1	OMS	CN	M−H	3.03	57
ZIF-7	Zn	380	0.16	8.0		ES	π−H	2.66	58
M_2(dobpdc)	Mg	3270	1.256	10.7					59
M_2(dobpdc)	Mn	2134	1.056	8.7					59
M_2(dobpdc)	Fe	2607	1.046	10.0	OMS	CN	M−H	2.44	59
M_2(dobpdc)	Co	2255	1.03	11.3					59
M_2(dobpdc)	Ni	2059	1.032	12.0					59
M_2(dobpdc)	Zn	1873	0.998	8.4					59
M_2(dobdc)	Mg	1800	1.648	10.6	OMS	CN	M−H	2.45	59
M_2(dobdc)	Mn	1447	1.316	8.8					59
M_2(dobdc)	Co	1341	1.282	10.7	OMS	CN	M−H	2.32	59
M_2(dobdc)	Ni	1218	1.284	12.9	OMS	CN	M−H	2.201	59
M_2(dobdc)	Zn	747	1.23	8.4					59
Fe_2(dobdc)	Fe	1360	2.15	9.7	OMS	CN	M−H	2.47	60
$Fe_2(O_2)$(dobdc)	Fe	1150	1.83	10.0	OMS	CN	M−H	2.53	60

M_2(m-dobdc)	Mn	1741	1.84	10.3					61
M_2(m-dobdc)	Fe	1624	2.00	11.1					61
M_2(m-dobdc)	Co	1504	1.76	11.5	OMS	CN	M−H	2.23	61
M_2(m-dobdc)	Ni	1592	2.21	12.3	OMS	CN	M−H	2.18	61

Table S5 The reported adsorption performance and the host-guest interaction of MOFs (SO_2)

Material	Metal	BET (m^2/g)	Uptake at 273 K, 1 bar (mmol/g)	Q_{st} (kJ/mol)	Active site	Main Interaction	Primary Interaction	Distance (Å)	Reference
MFM-305-Me	Al	256	5.29	33	CH_3	HB	CH−O	2.49	47
MFM-305	Al	779	9.05	41		ES	N−S	2.78	47
MFM-300(In)	In	1071	8.28 (298 K)	37.2	OH	HB	OH−O	3.17	1
MFM-520	Zn	313	3.75	96		HB	CH−O	2.33	32
M-gallate	Mg	576	8.15	60	OH	HB	OH−O	1.803	62
M-gallate	Co	494	8.82	55	OH	HB	OH−O	2.198	62
M-gallate	Ni	455	7.83	55	OH	HB	OH−O	1.979	62
MFM-601	Zr	3644	16.9	38	OH	HB	OH−O	2.53	62
MFM-300(Al)	Al	1370	8.1	N/A	OH	HB	OH−O	2.376	38
MFM-170	Cu	2408	19.4	35.4	OMS	CN	M−O	2.28	63
MFM-300($Al_{0.67}Cr_{0.33}$)	Al, Cr	1305	8.59	48.45	OH	HB	OH−O	2.333	49
MFM-300Cr	Cr	1360	10	43.2	OH	HB	OH−O	2.526	49

Table S6 The reported adsorption performance and the host-guest interaction of MOFs (C_2H_2)

Material	Metal	BET (cm^2/g)	Uptake (298 K, 1 bar, cc/g)	Q_{st} (kJ/mol)	Active site	Main Interaction	Primary interaction	Distance (Å)	Reference
SIFSIX-14-Cu-i	Cu	612	116	56	SiF_6	HB	F−HC	1.921	64
CPL-1	Cu	400	42	42.5		HB	O−HC	2.57/2.64	65
MFM-127	Cu	1557	194	N/A		HB	N−HC	2.54	66
NKMOF-1-Ni	Ni	382	61	60.3		HB	S−HC	3.86/3.92	67
Fe(pyz)Ni(CN)$_4$	Fe, Ni	383	96	32.8		π-π Bond	π−π	3.552	68
SIFSIX-Cu-TPA	Cu	1330	185	39.1	SiF_6	HB	F−HC	2.00	9
ATC-Cu	Cu	600	112	79.1	OMS	CN	M−π	2.90	69
MFM-520	Zn	313	69.2	60		HB	O−HC	2.72	70
Mg(HCOO)$_2$	Mg	284	65.7	38.5		HB	O−HC	2.51-2.66	71
Mn(HCOO)$_2$	Mn	297	51.2	38.5		HB	O−HC	2.51-2.66	71
APPT-Cd-ClO_4^-	Cd		39.3	28.5	ClO_4	HB	O−HC	2.248	72
Cu(INAIP)	Cu	N/A	N/A	N/A		HB	O−HC	2.647	73
SBMOF-1	Ca	145	30.4	34.8		H-π Bond	π−HC	3.23	74
SBMOF-2	Ca	195	64.7	55.4		H-π Bond	π−HC	3.11	74
Cu(etz)	Cu	N/A	70	32.2		HB	N−HC	2.50	75
HKUST-1	Cu	2095	201	N/A	OMS	CN	M−π	2.62	75
PCP-N	Fe	N/A	55	32.8		HB	N−HC	2.84	61
MFM-102-NO_2	Cu	2893	292	33	OMS	CN	M−π	2.93	76
Cu[Ni(2,3-pyrazinedithiolate)$_2$]	Cu, Ni	426	47.5			π-π Bond	π−π	3.41	77
MFM-300(Al)	Al	1370	142	31	OH	H-π Bond	OH−π	3.26	78
MFM-188	Cu	2568	232	32.5	OMS	CN	M−π	2.37	39
MFM-300(V^{III})	V	1892	163	32	OH	H-π Bond	OH−π	3.02	79
MFM-300(V^{IV})	V	1565	150	67		HB	O−HC	2.34	79

MFM-300 ($Al_{0.67}Cr_{0.33}$)	Al, Cr	1305	183	31.8		H-π Bond	π−HC	3.01	49
MFM-300Cr	Cr	1360	160	38.5		H-π Bond	π−HC	3.51	49
Fe_2(dobdc)	Fe	1350	175	47	OMS	CN	M−π	2.48	80
SIFSIX-1-Cu	Cu	1337	190	44.6	SiF_6	HB	F−HC	2.017	81

2. References

1) Savage, M.; Cheng, Y.; Easun, T. L.; Eyley, J. E.; Argent, S. P.; Warren, M. R.; Lewis, W.; Murray, C.; Tang, C. C.; Frogley, M. D.; Cinque, G.; Sun, J.; Rudić, S.; Murden, R. T.; Benham, M. J.; Fitch, A. N.; Blake, A. J.; Ramirez-Cuesta, A. J.; Yang, S.; Schröder, M. Selective Adsorption of Sulfur Dioxide in a Robust Metal–Organic Framework Material. *Adv. Mater.* **2016**, *28*, 8705–8711. https://doi.org/10.1002/adma.201602338.

2) Wriedt, M.; Sculley, J. P.; Yakovenko, A. A.; Ma, Y.; Halder, G. J.; Balbuena, P. B.; Zhou, H.-C. Low-Energy Selective Capture of Carbon Dioxide by a Pre-Designed Elastic Single-Molecule Trap. *Angew. Chem. Int. Ed Engl.* **2012**, *51*, 9804–9808. https://doi.org/10.1002/anie.201202992.

3) Plonka, A. M.; Banerjee, D.; Woerner, W. R.; Zhang, Z.; Nijem, N.; Chabal, Y. J.; Li, J.; Parise, J. B. Mechanism of Carbon Dioxide Adsorption in a Highly Selective Coordination Network Supported by Direct Structural Evidence. *Angew. Chem. Int. Ed Engl.* **2013**, *52*, 1692–1695. https://doi.org/10.1002/anie.201207808.

4) Bezuidenhout, C. X.; Smith, V. J.; Bhatt, P. M.; Esterhuysen, C.; Barbour, L. J. Extreme Carbon Dioxide Sorption Hysteresis in Open-Channel Rigid Metal-Organic Frameworks. *Angew. Chem. Int. Ed Engl.* **2015**, *54*, 2079–2083. https://doi.org/10.1002/anie.201408933.

5) Cheng, W.; Compton, R. G. Investigation of Single-Drug-Encapsulating Liposomes Using the Nano-Impact Method. *Angew. Chem. Int. Ed Engl.* **2014**, *53*, 13928–13930. https://doi.org/10.1002/anie.201408934.

6) Sotelo, J.; Woodall, C. H.; Allan, D. R.; Gregoryanz, E.; Howie, R. T.; Kamenev, K. V.; Probert, M. R.; Wright, P. A.; Moggach, S. A. Locating Gases in Porous Materials: Cryogenic Loading of Fuel-Related Gases into a Sc-Based Metal-Organic Framework under Extreme Pressures. *Angew. Chem. Int. Ed Engl.* **2015**, *54*, 13332–13336. https://doi.org/10.1002/anie.201506250.

7) Lama, P.; Aggarwal, H.; Bezuidenhout, C. X.; Barbour, L. J. Giant Hysteretic Sorption of CO2 : In Situ Crystallographic Visualization of Guest Binding within a Breathing Framework at 298 K. *Angew. Chem. Int. Ed Engl.* **2016**, *55*, 13271–13275. https://doi.org/10.1002/anie.201607076.

8) Wang, Z.-S.; Li, M.; Peng, Y.-L.; Zhang, Z.; Chen, W.; Huang, X.-C. An Ultrastable Metal Azolate Framework with Binding Pockets for Optimal Carbon Dioxide Capture. *Angew. Chem. Int. Ed Engl.* **2019**, *58*, 16071–16076. https://doi.org/10.1002/anie.201909046.

9) Li, H.; Liu, C.; Chen, C.; Di, Z.; Yuan, D.; Pang, J.; Wei, W.; Wu, M.; Hong, M. An Unprecedented Pillar-Cage Fluorinated Hybrid Porous Framework with Highly Efficient Acetylene Storage and Separation. *Angew. Chem. Int. Ed Engl.* **2021**, *60*, 7547–7552. https://doi.org/10.1002/anie.202013988.

10) Xie, Y.; Cui, H.; Wu, H.; Lin, R.-B.; Zhou, W.; Chen, B. Electrostatically Driven Selective Adsorption of Carbon Dioxide over Acetylene in an Ultramicroporous Material. *Angew. Chem. Int. Ed Engl.* **2021**, *60*, 9604–9609. https://doi.org/10.1002/anie.202100584.

11) Han, Z.; Li, J.; Lu, W.; Wang, K.; Chen, Y.; Zhang, X.; Lin, L.; Han, X.; Teat, S. J.; Frogley, M. D.; Yang, S.; Shi, W.; Cheng, P. A $\{Ni_{12}\}$-Wheel-Based Metal-Organic

Framework for Coordinative Binding of Sulphur Dioxide and Nitrogen Dioxide. *Angew. Chem. Int. Ed Engl.* **2022**, *61*, e202115585. https://doi.org/10.1002/anie.202115585.

12) Hu, X.-L.; Gong, Q.-H.; Zhong, R.-L.; Wang, X.-L.; Qin, C.; Wang, H.; Li, J.; Shao, K.-Z.; Su, Z.-M. Evidence of Amine-CO_2 Interactions in Two Pillared-Layer MOFs Probed by X-Ray Crystallography. *Chemistry* **2015**, *21*, 7238–7244. https://doi.org/10.1002/chem.201406495.

13) Yao, Z.; Chen, Y.; Liu, L.; Wu, X.; Xiong, S.; Zhang, Z.; Xiang, S. Direct Evidence of CO_2 Capture under Low Partial Pressure on a Pillared Metal-Organic Framework with Improved Stabilization through Intramolecular Hydrogen Bonding. *ChemPlusChem* **2016**, *81*, 850–856. https://doi.org/10.1002/cplu.201600156.

14) Couck, S.; Gobechiya, E.; Kirschhock, C. E. A.; Serra-Crespo, P.; Juan-Alcañiz, J.; Martinez Joaristi, A.; Stavitski, E.; Gascon, J.; Kapteijn, F.; Baron, G. V.; Denayer, J. F. M. Adsorption and Separation of Light Gases on an Amino-Functionalized Metal-Organic Framework: An Adsorption and in Situ XRD Study. *ChemSusChem* **2012**, *5*, 740–750. https://doi.org/10.1002/cssc.201100378.

15) Zhang, X.-W.; Zhou, D.-D.; Zhang, J.-P. Tuning the Gating Energy Barrier of Metal-Organic Framework for Molecular Sieving. *Chem* **2021**, *7*, 1006–1019. https://doi.org/10.1016/j.chempr.2020.12.025.

16) Forse, A. C.; Colwell, K. A.; Gonzalez, M. I.; Benders, S.; Torres-Gavosto, R. M.; Blümich, B.; Reimer, J. A.; Long, J. R. Influence of Pore Size on Carbon Dioxide Diffusion in Two Isoreticular Metal–Organic Frameworks. *Chem. Mater.* **2020**, *32*, 3570–3576. https://doi.org/10.1021/acs.chemmater.0c00745.

17) Chen, S.; Lucier, B. E. G.; Boyle, P. D.; Huang, Y. Understanding the Fascinating Origins of CO_2 Adsorption and Dynamics in MOFs. *Chem. Mater.* **2016**, *28*, 5829–5846. https://doi.org/10.1021/acs.chemmater.6b02239.

18) Chen, M.; Chen, S.; Chen, W.; Lucier, B. E. G.; Zhang, Y.; Zheng, A.; Huang, Y. Analyzing Gas Adsorption in an Amide-Functionalized Metal Organic Framework: Are the Carbonyl or Amine Groups Responsible? *Chem. Mater.* **2018**, *30*, 3613–3617. https://doi.org/10.1021/acs.chemmater.8b00681.

19) Mon, M.; Bruno, R.; Tiburcio, E.; Grau-Atienza, A.; Sepúlveda-Escribano, A.; Ramos-Fernandez, E. V.; Fuoco, A.; Esposito, E.; Monteleone, M.; Jansen, J. C.; Cano, J.; Ferrando-Soria, J.; Armentano, D.; Pardo, E. Efficient Gas Separation and Transport Mechanism in Rare Hemilabile Metal–Organic Framework. *Chem. Mater.* **2019**, *31*, 5856–5866. https://doi.org/10.1021/acs.chemmater.9b01995.

20) Asgari, M.; Semino, R.; Schouwink, P. A.; Kochetygov, I.; Tarver, J.; Trukhina, O.; Krishna, R.; Brown, C. M.; Ceriotti, M.; Queen, W. L. Understanding How Ligand Functionalization Influences CO_2 and N_2 Adsorption in a Sodalite Metal-Organic Framework. *Chem. Mater.* **2020**, *32*, 1526–1536. https://doi.org/10.1021/acs.chemmater.9b04631.

21) Miller, R. G.; Warren, M. R.; Allan, D. R.; Brooker, S. Direct Crystallographic Observation of CO_2 Captured in Zig Zag Channels of a Copper(I) Metal-Organic

Framework. *Inorg. Chem.* **2020**, *59*, 6376–6381. https://doi.org/10.1021/acs.inorgchem.0c00471.

22) Krap, C. P.; Newby, R.; Dhakshinamoorthy, A.; García, H.; Cebula, I.; Easun, T. L.; Savage, M.; Eyley, J. E.; Gao, S.; Blake, A. J.; Lewis, W.; Beton, P. H.; Warren, M. R.; Allan, D. R.; Frogley, M. D.; Tang, C. C.; Cinque, G.; Yang, S.; Schröder, M. Enhancement of CO_2 Adsorption and Catalytic Properties by Fe-Doping of $[Ga_2(OH)_2(L)]$ (H_4L = Biphenyl-3,3',5,5'-Tetracarboxylic Acid), MFM-300(Ga_2). *Inorg. Chem.* **2016**, *55*, 1076–1088. https://doi.org/10.1021/acs.inorgchem.5b02108.

23) Ye, Y.; Xiong, S.; Wu, X.; Zhang, L.; Li, Z.; Wang, L.; Ma, X.; Chen, Q.-H.; Zhang, Z.; Xiang, S. Microporous Metal-Organic Framework Stabilized by Balanced Multiple Host-Couteranion Hydrogen-Bonding Interactions for High-Density CO_2 Capture at Ambient Conditions. *Inorg. Chem.* **2016**, *55*, 292–299. https://doi.org/10.1021/acs.inorgchem.5b02316.

24) Bolotov, V. A.; Kovalenko, K. A.; Samsonenko, D. G.; Han, X.; Zhang, X.; Smith, G. L.; McCormick, L. J.; Teat, S. J.; Yang, S.; Lennox, M. J.; Henley, A.; Besley, E.; Fedin, V. P.; Dybtsev, D. N.; Schröder, M. Enhancement of CO_2 Uptake and Selectivity in a Metal-Organic Framework by the Incorporation of Thiophene Functionality. *Inorg. Chem.* **2018**, *57*, 5074–5082. https://doi.org/10.1021/acs.inorgchem.8b00138.

25) Heymans, N.; Bourrelly, S.; Normand, P.; Bloch, E.; Mkhadder, H.; Cooper, L.; Gorman, M.; Bouzidi, I.; Guillou, N.; De Weireld, G.; Devic, T. Small-Pore Gallates MOFs for Environmental Applications: Sorption Behaviors and Structural Elucidation of Their High Affinity for CO_2. *J. Phys. Chem. C Nanomater. Interfaces* **2020**, *124*, 3188–3195. https://doi.org/10.1021/acs.jpcc.9b11535.

26) Jiang, M.; Li, B.; Cui, X.; Yang, Q.; Bao, Z.; Yang, Y.; Wu, H.; Zhou, W.; Chen, B.; Xing, H. Controlling Pore Shape and Size of Interpenetrated Anion-Pillared Ultramicroporous Materials Enables Molecular Sieving of CO_2 Combined with Ultrahigh Uptake Capacity. *ACS Appl. Mater. Interfaces* **2018**, *10*, 16628–16635. https://doi.org/10.1021/acsami.8b03358.

27) Han, L.; Pham, T.; Zhuo, M.; Forrest, K. A.; Suepaul, S.; Space, B.; Zaworotko, M. J.; Shi, W.; Chen, Y.; Cheng, P.; Zhang, Z. Molecular Sieving and Direct Visualization of CO_2 in Binding Pockets of an Ultramicroporous Lanthanide Metal-Organic Framework Platform. *ACS Appl. Mater. Interfaces* **2019**, *11*, 23192–23197. https://doi.org/10.1021/acsami.9b04619.

28) Wu, B.; Wong, Y. T. A.; Lucier, B. E. G.; Boyle, P. D.; Huang, Y. Exploring Host-Guest Interactions in the α-$Zn_3(HCOO)_6$ Metal-Organic Framework. *ACS Omega* **2019**, *4*, 4000–4011. https://doi.org/10.1021/acsomega.8b03623.

29) Chae, S. H.; Kim, H.-C.; Lee, Y. S.; Huh, S.; Kim, S.-J.; Kim, Y.; Lee, S. J. Thermally Robust 3-D Co-DpyDtolP-MOF with Hexagonally Oriented Micropores: Formation of Polyiodine Chains in a MOF Single Crystal. *Cryst. Growth Des.* **2015**, *15*, 268–277. https://doi.org/10.1021/cg501324r.

30) Bon, V.; Senkovska, I.; Wallacher, D.; Többens, D. M.; Zizak, I.; Feyerherm, R.; Mueller, U.; Kaskel, S. In Situ Observation of Gating Phenomena in the Flexible Porous Coordination Polymer $Zn_2(BPnDC)_2(Bpy)$ (SNU-9) in a Combined Diffraction and Gas

Adsorption Experiment. *Inorg. Chem.* **2014**, *53*, 1513–1520. https://doi.org/10.1021/ic4024844.

31) Liao, P.-Q.; Zhou, D.-D.; Zhu, A.-X.; Jiang, L.; Lin, R.-B.; Zhang, J.-P.; Chen, X.-M. Strong and Dynamic CO_2 Sorption in a Flexible Porous Framework Possessing Guest Chelating Claws. *J. Am. Chem. Soc.* **2012**, *134*, 17380–17383. https://doi.org/10.1021/ja3073512.

32) Zhang, J.-P.; Chen, X.-M. Optimized Acetylene/Carbon Dioxide Sorption in a Dynamic Porous Crystal. *J. Am. Chem. Soc.* **2009**, *131*, 5516–5521. https://doi.org/10.1021/ja8089872.

33) Li, J.; Zhou, Z.; Han, X.; Zhang, X.; Yan, Y.; Li, W.; Smith, G. L.; Cheng, Y.; McCormick McPherson, L. J.; Teat, S. J.; Frogley, M. D.; Rudić, S.; Ramirez-Cuesta, A. J.; Blake, A. J.; Sun, J.; Schröder, M.; Yang, S. Guest-Controlled Incommensurate Modulation in a Meta-Rigid Metal-Organic Framework Material. *J. Am. Chem. Soc.* **2020**, *142*, 19189–19197. https://doi.org/10.1021/jacs.0c08794.

34) Ma, Y.; Matsuda, R.; Sato, H.; Hijikata, Y.; Li, L.; Kusaka, S.; Foo, M.; Xue, F.; Akiyama, G.; Yuan, R.; Kitagawa, S. A Convenient Strategy for Designing a Soft Nanospace: An Atomic Exchange in a Ligand with Isostructural Frameworks. *J. Am. Chem. Soc.* **2015**, *137*, 15825–15832. https://doi.org/10.1021/jacs.5b09666.

35) Bhatt, P. M.; Belmabkhout, Y.; Cadiau, A.; Adil, K.; Shekhah, O.; Shkurenko, A.; Barbour, L. J.; Eddaoudi, M. A Fine-Tuned Fluorinated MOF Addresses the Needs for Trace CO_2 Removal and Air Capture Using Physisorption. *J. Am. Chem. Soc.* **2016**, *138*, 9301–9307. https://doi.org/10.1021/jacs.6b05345.

36) Benson, O.; da Silva, I.; Argent, S. P.; Cabot, R.; Savage, M.; Godfrey, H. G. W.; Yan, Y.; Parker, S. F.; Manuel, P.; Lennox, M. J.; Mitra, T.; Easun, T. L.; Lewis, W.; Blake, A. J.; Besley, E.; Yang, S.; Schröder, M. Amides Do Not Always Work: Observation of Guest Binding in an Amide-Functionalized Porous Metal–Organic Framework. *J. Am. Chem. Soc.* **2016**, *138*, 14828–14831. https://doi.org/10.1021/jacs.6b08059.

37) Forse, A. C.; Gonzalez, M. I.; Siegelman, R. L.; Witherspoon, V. J.; Jawahery, S.; Mercado, R.; Milner, P. J.; Martell, J. D.; Smit, B.; Blümich, B.; Long, J. R.; Reimer, J. A. Unexpected Diffusion Anisotropy of Carbon Dioxide in the Metal–Organic Framework Zn_2(Dobpdc). *J. Am. Chem. Soc.* **2018**, *140*, 1663–1673. https://doi.org/10.1021/jacs.7b09453.

38) Carter, J. H.; Han, X.; Moreau, F. Y.; da Silva, I.; Nevin, A.; Godfrey, H. G. W.; Tang, C. C.; Yang, S.; Schröder, M. Exceptional Adsorption and Binding of Sulfur Dioxide in a Robust Zirconium-Based Metal-Organic Framework. *J. Am. Chem. Soc.* **2018**, *140*, 15564–15567. https://doi.org/10.1021/jacs.8b08433.

39) Yang, S.; Sun, J.; Ramirez-Cuesta, A. J.; Callear, S. K.; David, W. I. F.; Anderson, D. P.; Newby, R.; Blake, A. J.; Parker, J. E.; Tang, C. C.; Schröder, M. Selectivity and Direct Visualization of Carbon Dioxide and Sulfur Dioxide in a Decorated Porous Host. *Nat. Chem.* **2012**, *4*, 887–894. https://doi.org/10.1038/nchem.1457.

40) Moreau, F.; da Silva, I.; Al Smail, N. H.; Easun, T. L.; Savage, M.; Godfrey, H. G. W.; Parker, S. F.; Manuel, P.; Yang, S.; Schröder, M. Unravelling Exceptional Acetylene and

Carbon Dioxide Adsorption within a Tetra-Amide Functionalized Metal-Organic Framework. *Nat. Commun.* **2017**, *8*, 14085. https://doi.org/10.1038/ncomms14085.

41) Lu, Z.; Godfrey, H. G. W.; da Silva, I.; Cheng, Y.; Savage, M.; Tuna, F.; McInnes, E. J. L.; Teat, S. J.; Gagnon, K. J.; Frogley, M. D.; Manuel, P.; Rudić, S.; Ramirez-Cuesta, A. J.; Easun, T. L.; Yang, S.; Schröder, M. Modulating Supramolecular Binding of Carbon Dioxide in a Redox-Active Porous Metal-Organic Framework. *Nat. Commun.* **2017**, *8*, 14212. https://doi.org/10.1038/ncomms14212.

42) Xiang, S.; He, Y.; Zhang, Z.; Wu, H.; Zhou, W.; Krishna, R.; Chen, B. Microporous Metal-Organic Framework with Potential for Carbon Dioxide Capture at Ambient Conditions. *Nat. Commun.* **2012**, *3*, 954. https://doi.org/10.1038/ncomms1956.

43) Liao, P.-Q.; Zhang, W.-X.; Zhang, J.-P.; Chen, X.-M. Efficient Purification of Ethene by an Ethane-Trapping Metal-Organic Framework. *Nat. Commun.* **2015**, *6*, 8697. https://doi.org/10.1038/ncomms9697.

44) Wu, P.; Li, Y.; Zheng, J.-J.; Hosono, N.; Otake, K.-I.; Wang, J.; Liu, Y.; Xia, L.; Jiang, M.; Sakaki, S.; Kitagawa, S. Carbon Dioxide Capture and Efficient Fixation in a Dynamic Porous Coordination Polymer. *Nat. Commun.* **2019**, *10*, 4362. https://doi.org/10.1038/s41467-019-12414-z.

45) Zhao, P.; Lampronti, G. I.; Lloyd, G. O.; Suard, E.; Redfern, S. A. T. Direct Visualisation of Carbon Dioxide Adsorption in Gate-Opening Zeolitic Imidazolate Framework ZIF-7. *J. Mater. Chem. A Mater. Energy Sustain.* **2014**, *2*, 620–623. https://doi.org/10.1039/c3ta13981f.

46) Queen, W. L.; Hudson, M. R.; Bloch, E. D.; Mason, J. A.; Gonzalez, M. I.; Lee, J. S.; Gygi, D.; Howe, J. D.; Lee, K.; Darwish, T. A.; James, M.; Peterson, V. K.; Teat, S. J.; Smit, B.; Neaton, J. B.; Long, J. R.; Brown, C. M. Comprehensive Study of Carbon Dioxide Adsorption in the Metal–Organic Frameworks M_2(Dobdc) (M = Mg, Mn, Fe, Co, Ni, Cu, Zn). *Chem. Sci.* **2014**, *5*, 4569–4581. https://doi.org/10.1039/c4sc02064b.

47) Shin, J. W.; Jeong, A. R.; Jeoung, S.; Moon, H. R.; Komatsumaru, Y.; Hayami, S.; Moon, D.; Min, K. S. Three-Dimensional Iron(Ii) Porous Coordination Polymer Exhibiting Carbon Dioxide-Dependent Spin Crossover. *Chem. Commun. (Camb.)* **2018**, *54*, 4262–4265. https://doi.org/10.1039/c8cc00678d.

48) Li, L.; da Silva, I.; Kolokolov, D. I.; Han, X.; Li, J.; Smith, G.; Cheng, Y.; Daemen, L. L.; Morris, C. G.; Godfrey, H. G. W.; Jacques, N. M.; Zhang, X.; Manuel, P.; Frogley, M. D.; Murray, C. A.; Ramirez-Cuesta, A. J.; Cinque, G.; Tang, C. C.; Stepanov, A. G.; Yang, S.; Schroder, M. Post-Synthetic Modulation of the Charge Distribution in a Metal-Organic Framework for Optimal Binding of Carbon Dioxide and Sulfur Dioxide. *Chem. Sci.* **2019**, *10*, 1472–1482. https://doi.org/10.1039/c8sc01959b.

49) Humby, J. D.; Benson, O.; Smith, G. L.; Argent, S. P.; da Silva, I.; Cheng, Y.; Rudić, S.; Manuel, P.; Frogley, M. D.; Cinque, G.; Saunders, L. K.; Vitórica-Yrezábal, I. J.; Whitehead, G. F. S.; Easun, T. L.; Lewis, W.; Blake, A. J.; Ramirez-Cuesta, A. J.; Yang, S.; Schröder, M. Host-Guest Selectivity in a Series of Isoreticular Metal-Organic Frameworks: Observation of Acetylene-to-Alkyne and Carbon Dioxide-to-Amide Interactions. *Chem. Sci.* **2019**, *10*, 1098–1106. https://doi.org/10.1039/c8sc03622e.

50) Briggs, L.; Newby, R.; Han, X.; Morris, C. G.; Savage, M.; Krap, C. P.; Easun, T. L.; Frogley, M. D.; Cinque, G.; Murray, C. A.; Tang, C. C.; Sun, J.; Yang, S.; Schröder, M. Binding and Separation of CO_2, SO_2 and C_2H_2 in Homo- and Hetero-Metallic Metal–Organic Framework Materials. *J. Mater. Chem. A Mater. Energy Sustain.* **2021**, *9*, 7190–7197. https://doi.org/10.1039/d1ta00687h.

51) Kubota, Y.; Takata, M.; Matsuda, R.; Kitaura, R.; Kitagawa, S.; Kato, K.; Sakata, M.; Kobayashi, T. C. Direct Observation of Hydrogen Molecules Adsorbed onto a Microporous Coordination Polymer. *Angew. Chem. Int. Ed Engl.* **2005**, *44*, 920–923. https://doi.org/10.1002/anie.200461895.

52) Bloch, E. D.; Queen, W. L.; Hudson, M. R.; Mason, J. A.; Xiao, D. J.; Murray, L. J.; Flacau, R.; Brown, C. M.; Long, J. R. Hydrogen Storage and Selective, Reversible O_2 Adsorption in a Metal-Organic Framework with Open Chromium(II) Sites. *Angew. Chem. Int. Ed Engl.* **2016**, *55*, 8605–8609. https://doi.org/10.1002/anie.201602950.

53) Asgari, M.; Semino, R.; Schouwink, P.; Kochetygov, I.; Trukhina, O.; Tarver, J. D.; Bulut, S.; Yang, S.; Brown, C. M.; Ceriotti, M.; Queen, W. L. An In-situ Neutron Diffraction and DFT Study of Hydrogen Adsorption in a Sodalite-type Metal–Organic Framework, Cu-BTTri: An in-Situ Neutron Diffraction and DFT Study of Hydrogen Adsorption in a Sodalite-Type Metal-Organic Framework, Cu-BTTri. *Eur. J. Inorg. Chem.* **2019**, *2019*, 1147–1154. https://doi.org/10.1002/ejic.201801253.

54) Yan, Y.; da Silva, I.; Blake, A. J.; Dailly, A.; Manuel, P.; Yang, S.; Schröder, M. High Volumetric Hydrogen Adsorption in a Porous Anthracene-Decorated Metal-Organic Framework. *Inorg. Chem.* **2018**, *57*, 12050–12055. https://doi.org/10.1021/acs.inorgchem.8b01607.

55) Barnett, B. R.; Evans, H. A.; Su, G. M.; Jiang, H. Z. H.; Chakraborty, R.; Banyeretse, D.; Hartman, T. J.; Martinez, M. B.; Trump, B. A.; Tarver, J. D.; Dods, M. N.; Funke, L. M.; Börgel, J.; Reimer, J. A.; Drisdell, W. S.; Hurst, K. E.; Gennett, T.; FitzGerald, S. A.; Brown, C. M.; Head-Gordon, M.; Long, J. R. Observation of an Intermediate to H2 Binding in a Metal-Organic Framework. *J. Am. Chem. Soc.* **2021**, *143*, 14884–14894. https://doi.org/10.1021/jacs.1c07223.

56) Savage, M.; da Silva, I.; Johnson, M.; Carter, J. H.; Newby, R.; Suyetin, M.; Besley, E.; Manuel, P.; Rudić, S.; Fitch, A. N.; Murray, C.; David, W. I. F.; Yang, S.; Schröder, M. Observation of Binding and Rotation of Methane and Hydrogen within a Functional Metal-Organic Framework. *J. Am. Chem. Soc.* **2016**, *138*, 9119–9127. https://doi.org/10.1021/jacs.6b01323.

57) Spencer, E. C.; Howard, J. A. K.; McIntyre, G. J.; Rowsell, J. L. C.; Yaghi, O. M. Determination of the Hydrogen Absorption Sites in Zn_4O(1,4-Benzenedicarboxylate) by Single Crystal Neutron Diffraction. *Chem. Commun. (Camb.)* **2006**, 278–280. https://doi.org/10.1039/b511941c.

58) Rosnes, M. H.; Opitz, M.; Frontzek, M.; Lohstroh, W.; Embs, J. P.; Georgiev, P. A.; Dietzel, P. D. C. Intriguing Differences in Hydrogen Adsorption in CPO-27 Materials Induced by Metal Substitution. *J. Mater. Chem. A Mater. Energy Sustain.* **2015**, *3*, 4827–4839. https://doi.org/10.1039/c4ta05794e.

59) Klein, R. A.; Shulda, S.; Parilla, P. A.; Le Magueres, P.; Richardson, R. K.; Morris, W.; Brown, C. M.; McGuirk, C. M. Structural Resolution and Mechanistic Insight into Hydrogen Adsorption in Flexible ZIF-7. *Chem. Sci.* **2021**, *12*, 15620–15631. https://doi.org/10.1039/d1sc04618g.

60) Gygi, D.; Bloch, E. D.; Mason, J. A.; Hudson, M. R.; Gonzalez, M. I.; Siegelman, R. L.; Darwish, T. A.; Queen, W. L.; Brown, C. M.; Long, J. R. Hydrogen Storage in the Expanded Pore Metal–Organic Frameworks M_2(Dobpdc) (M = Mg, Mn, Fe, Co, Ni, Zn). *Chem. Mater.* **2016**, *28*, 1128–1138. https://doi.org/10.1021/acs.chemmater.5b04538.

61) Queen, W. L.; Bloch, E. D.; Brown, C. M.; Hudson, M. R.; Mason, J. A.; Murray, L. J.; Ramirez-Cuesta, A. J.; Peterson, V. K.; Long, J. R. Hydrogen Adsorption in the Metal-Organic Frameworks Fe_2(Dobdc) and $Fe_2(O_2)$(Dobdc). *Dalton Trans.* **2012**, *41*, 4180–4187. https://doi.org/10.1039/c2dt12138g.

62) Kapelewski, M. T.; Geier, S. J.; Hudson, M. R.; Stück, D.; Mason, J. A.; Nelson, J. N.; Xiao, D. J.; Hulvey, Z.; Gilmour, E.; FitzGerald, S. A.; Head-Gordon, M.; Brown, C. M.; Long, J. R. M_2(m-Dobdc) (M = Mg, Mn, Fe, Co, Ni) Metal-Organic Frameworks Exhibiting Increased Charge Density and Enhanced H2 Binding at the Open Metal Sites. *J. Am. Chem. Soc.* **2014**, *136*, 12119–12129. https://doi.org/10.1021/ja506230r.

63) Chen, F.; Lai, D.; Guo, L.; Wang, J.; Zhang, P.; Wu, K.; Zhang, Z.; Yang, Q.; Yang, Y.; Chen, B.; Ren, Q.; Bao, Z. Deep Desulfurization with Record SO_2 Adsorption on the Metal-Organic Frameworks. *J. Am. Chem. Soc.* **2021**, *143*, 9040–9047. https://doi.org/10.1021/jacs.1c02176.

64) Smith, G. L.; Eyley, J. E.; Han, X.; Zhang, X.; Li, J.; Jacques, N. M.; Godfrey, H. G. W.; Argent, S. P.; McCormick McPherson, L. J.; Teat, S. J.; Cheng, Y.; Frogley, M. D.; Cinque, G.; Day, S. J.; Tang, C. C.; Easun, T. L.; Rudić, S.; Ramirez-Cuesta, A. J.; Yang, S.; Schröder, M. Reversible Coordinative Binding and Separation of Sulfur Dioxide in a Robust Metal-Organic Framework with Open Copper Sites. *Nat. Mater.* **2019**, *18*, 1358–1365. https://doi.org/10.1038/s41563-019-0495-0.

65) Li, B.; Cui, X.; O'Nolan, D.; Wen, H.-M.; Jiang, M.; Krishna, R.; Wu, H.; Lin, R.-B.; Chen, Y.-S.; Yuan, D.; Xing, H.; Zhou, W.; Ren, Q.; Qian, G.; Zaworotko, M. J.; Chen, B. An Ideal Molecular Sieve for Acetylene Removal from Ethylene with Record Selectivity and Productivity. *Adv. Mater.* **2017**, *29*, 1704210. https://doi.org/10.1002/adma.201704210.

66) Kubota, Y.; Takata, M.; Matsuda, R.; Kitaura, R.; Kitagawa, S.; Kobayashi, T. C. Metastable Sorption State of a Metal-Organic Porous Material Determined by in Situ Synchrotron Powder Diffraction. *Angew. Chem. Int. Ed Engl.* **2006**, *45*, 4932–4936. https://doi.org/10.1002/anie.200600976.

67) Xiang, S.; Zhou, W.; Zhang, Z.; Green, M. A.; Liu, Y.; Chen, B. Open Metal Sites within Isostructural Metal-Organic Frameworks for Differential Recognition of Acetylene and Extraordinarily High Acetylene Storage Capacity at Room Temperature. *Angew. Chem. Int. Ed Engl.* **2010**, *49*, 4615–4618. https://doi.org/10.1002/anie.201000094.

68) Peng, Y.-L.; Pham, T.; Li, P.; Wang, T.; Chen, Y.; Chen, K.-J.; Forrest, K. A.; Space, B.; Cheng, P.; Zaworotko, M. J.; Zhang, Z. Robust Ultramicroporous Metal-Organic

Frameworks with Benchmark Affinity for Acetylene. *Angew. Chem. Int. Ed Engl.* **2018**, *57*, 10971–10975. https://doi.org/10.1002/anie.201806732.

69) Gao, J.; Qian, X.; Lin, R.-B.; Krishna, R.; Wu, H.; Zhou, W.; Chen, B. Mixed Metal-Organic Framework with Multiple Binding Sites for Efficient C_2H_2/CO_2 Separation. *Angew. Chem. Int. Ed Engl.* **2020**, *59*, 4396–4400. https://doi.org/10.1002/anie.202000323.

70) Niu, Z.; Cui, X.; Pham, T.; Verma, G.; Lan, P. C.; Shan, C.; Xing, H.; Forrest, K. A.; Suepaul, S.; Space, B.; Nafady, A.; Al-Enizi, A. M.; Ma, S. A MOF-Based Ultra-Strong Acetylene Nano-Trap for Highly Efficient C_2H_2/CO_2 Separation. *Angew. Chem. Int. Ed Engl.* **2021**, *60*, 5283–5288. https://doi.org/10.1002/anie.202016225.

71) Li, J.; Han, X.; Kang, X.; Chen, Y.; Xu, S.; Smith, G. L.; Tillotson, E.; Cheng, Y.; McCormick McPherson, L. J.; Teat, S. J.; Rudić, S.; Ramirez-Cuesta, A. J.; Haigh, S. J.; Schröder, M.; Yang, S. Purification of Propylene and Ethylene by a Robust Metal-Organic Framework Mediated by Host-Guest Interactions. *Angew. Chem. Int. Ed Engl.* **2021**, *60*, 15541–15547. https://doi.org/10.1002/anie.202103936.

72) Samsonenko, D. G.; Kim, H.; Sun, Y.; Kim, G.-H.; Lee, H.-S.; Kim, K. Microporous Magnesium and Manganese Formates for Acetylene Storage and Separation. *Chem. Asian J.* **2007**, *2*, 484–488. https://doi.org/10.1002/asia.200600390.

73) Jin, G.-X.; Niu, X.; Wang, J.; Ma, J.-P.; Hu, T.-L.; Dong, Y.-B. APPT-Cd MOF: Acetylene Adsorption Mechanism and Its Highly Efficient Acetylene/Ethylene Separation at Room Temperature. *Chem. Mater.* **2018**, *30*, 7433–7437. https://doi.org/10.1021/acs.chemmater.8b03903.

74) Plonka, A. M.; Chen, X.; Wang, H.; Krishna, R.; Dong, X.; Banerjee, D.; Woerner, W. R.; Han, Y.; Li, J.; Parise, J. B. Light Hydrocarbon Adsorption Mechanisms in Two Calcium-Based Microporous Metal Organic Frameworks. *Chem. Mater.* **2016**, *28*, 1636–1646. https://doi.org/10.1021/acs.chemmater.5b03792.

75) Xiang, S.; Zhou, W.; Gallegos, J. M.; Liu, Y.; Chen, B. Exceptionally High Acetylene Uptake in a Microporous Metal-Organic Framework with Open Metal Sites. *J. Am. Chem. Soc.* **2009**, *131*, 12415–12419. https://doi.org/10.1021/ja904782h.

76) Duong, T. D.; Sapchenko, S. A.; da Silva, I.; Godfrey, H. G. W.; Cheng, Y.; Daemen, L. L.; Manuel, P.; Ramirez-Cuesta, A. J.; Yang, S.; Schröder, M. Optimal Binding of Acetylene to a Nitro-Decorated Metal-Organic Framework. *J. Am. Chem. Soc.* **2018**, *140*, 16006–16009. https://doi.org/10.1021/jacs.8b08504.

77) Aubrey, M. L.; Kapelewski, M. T.; Melville, J. F.; Oktawiec, J.; Presti, D.; Gagliardi, L.; Long, J. R. Chemiresistive Detection of Gaseous Hydrocarbons and Interrogation of Charge Transport in $Cu[Ni(2,3\text{-Pyrazinedithiolate})_2]$ by Gas Adsorption. *J. Am. Chem. Soc.* **2019**, *141*, 5005–5013. https://doi.org/10.1021/jacs.9b00654.

78) Yang, S.; Ramirez-Cuesta, A. J.; Newby, R.; Garcia-Sakai, V.; Manuel, P.; Callear, S. K.; Campbell, S. I.; Tang, C. C.; Schröder, M. Supramolecular Binding and Separation of Hydrocarbons within a Functionalized Porous Metal-Organic Framework. *Nat. Chem.* **2014**, *7*, 121–129. https://doi.org/10.1038/nchem.2114.

79) Lu, Z.; Godfrey, H. G. W.; da Silva, I.; Cheng, Y.; Savage, M.; Manuel, P.; Rudić, S.; Ramirez-Cuesta, A. J.; Yang, S.; Schröder, M. Direct Observation of Supramolecular

Binding of Light Hydrocarbons in Vanadium(III) and (IV) Metal–Organic Framework Materials. *Chem. Sci.* **2018**, *9*, 3401–3408. https://doi.org/10.1039/c8sc00330k.

80) Bloch, E. D.; Queen, W. L.; Krishna, R.; Zadrozny, J. M.; Brown, C. M.; Long, J. R. Hydrocarbon Separations in a Metal-Organic Framework with Open Iron(II) Coordination Sites. *Science* **2012**, *335*, 1606–1610. https://doi.org/10.1126/science.1217544.

81) Cui, X.; Chen, K.; Xing, H.; Yang, Q.; Krishna, R.; Bao, Z.; Wu, H.; Zhou, W.; Dong, X.; Han, Y.; Li, B.; Ren, Q.; Zaworotko, M. J.; Chen, B. Pore Chemistry and Size Control in Hybrid Porous Materials for Acetylene Capture from Ethylene. *Science* **2016**, *353*, 141–144. https://doi.org/10.1126/science.aaf2458.
